# The Fynbos and Succulent Karoo Biomes Do Not Have Exceptional Local Ant Richness

**DOI:** 10.1371/journal.pone.0031463

**Published:** 2012-03-02

**Authors:** Brigitte Braschler, Steven L. Chown, Kevin J. Gaston

**Affiliations:** 1 Biodiversity and Macroecology Group, Department of Animal and Plant Sciences, University of Sheffield, Sheffield, United Kingdom; 2 Centre for Invasion Biology, Department of Botany and Zoology, Stellenbosch University, Matieland, South Africa; Field Museum of Natural History, United States of America

## Abstract

**Background:**

The Fynbos (FB) and Succulent Karoo biomes (SKB) have high regional plant diversity despite relatively low productivity. Local diversity in the region varies but is moderate. For insects, previous work suggests that strict phytophages, but not other taxa, may have high regional richness. However, what has yet to be investigated is whether the local insect species richness of FB and SKB is unusual for a region of this productivity level at this latitude, and whether regional richness is also high. Here we determine whether this is the case for ants.

**Methodology/Principal Findings:**

We use species richness data from pitfall traps in the FB and SKB in the Western Cape Province, South Africa and a global dataset of local ant richness extracted from the literature. We then relate the globally derived values of local richness to two energy-related predictors—productive energy (NDVI) and temperature, and to precipitation, and compare the data from the FB and SKB with these relationships. We further compare our local richness estimates with that of similar habitats worldwide, and regional ant richness with estimates derived from other regions. The local ant species richness of the FB and SKB falls within the general global pattern relating ant richness to energy, and is similar to that in comparable habitats elsewhere. At a regional scale, the richness of ants across all of our sites is not exceptional by comparison with other regional estimates from across the globe.

**Conclusions/Significance:**

Local richness of ants in the FB and SKB is not exceptional by global standards. Initial analyses suggest that regional diversity is also not exceptional for the group. It seems unlikely that the mechanisms which have contributed to the development of extraordinarily high regional plant diversity in these biomes have had a strong influence on the ants.

## Introduction

Terrestrial biodiversity is not evenly distributed across the surface of the Earth. It is typically highest in the tropics, a pattern repeated in many taxonomic groups and one which has a considerable history [1]. A variety of mechanisms has been proposed to account for the latitudinal gradient in diversity, with energy variation being a primary contender as a consequence of its effect on extinction rates, speciation rates and the availability of rare resources [2],[3],[4],[5]. However, regional variation in diversification rates, often a result of differing geological or climatic histories, is also an important contributor to global species richness patterns [6],[7],[8].

The Fynbos Biome (FB) and the Succulent Karoo Biome (SKB) [9], located at the southern tip of Africa, are clear examples of regional exceptions to the general latitudinal pattern in species richness and its relationship with available energy. These biodiversity hotspots have exceptionally high regional plant diversity and endemism despite lying well outside the tropics and having relatively low productivity [10],[11],[12]. The diversity and endemism of plant species and genera of the FB are amongst the largest for any region of this size worldwide [13], while the SKB is one of only two entirely arid biodiversity hotspots [10],[11]. Consensus is growing that a variety of ecological and evolutionary processes have contributed to this high regional diversity, although energy availability is rarely counted among them [12],[14],[15],[16]. Indeed the plant species richness of the FB is about twice that predicted by models based on water-energy variables for regional floras [15],[16]. On the local scale, plant diversity in the FB and SKB varies between sub-regions and vegetation types, but overall is not exceptional, being comparable with similar habitat types worldwide [17],[18].

While the extraordinary regional plant diversity has attracted much interest, the region's invertebrate diversity is more poorly known [19]. Previously it has been suggested that insect diversity in the region is not high and may even be exceptionally low [20], perhaps partly as a consequence of low palatability of local plant species [21]. However, few studies have carefully explored this question, and comparisons with other areas of the globe are especially rare. In his qualitative review of the sparse literature on the topic, Giliomee [21] concluded that overall the arthropod diversity of the region is not exceptional when compared with other South African ecosystems, though the results differed among taxonomic groups. Comparisons with temperate or Mediterranean-type ecosystems worldwide similarly yielded mixed results depending on the taxonomic group, but for the most part did not support hypotheses of exceptional invertebrate diversity in the region. Focusing on a guild showing a close specialist association with plants, Wright & Samways [22] found high diversity for insect borer assemblages on *Protea* species in the FB — when compared with other South African biomes. Similarly a high diversity and high endemism was found for a phytophagous group, the Cicadellidae [23],[24]. Using sweep netting, Procheş & Cowling [25] found that insect diversity in the Fynbos is similar to or somewhat higher than that found in neighbouring South African biomes with similar plant diversity, at the local and biome scales, and noted that their work supported previous claims for a strong relationship between plant and insect species richness. Later work highlighted the role of similar responses in plants and insects to environmental factors as a mechanism underlying the plant-insect relationship at the broadest spatial scales [26]. In the Succulent Karoo, monkey beetle (Scarabaeidae) diversity shows high regional richness and high turnover, apparently associated with high plant turnover [27].

Combined the few existing studies suggest that those groups of insects that are directly dependent on plant diversity may have a high regional richness, but that the same might not be true for other taxa including less specialised herbivores. However, what is not clear, and indeed has yet to be investigated, is whether the local insect species richness of FB and SKB is unusual for a region of this productivity level at this latitude. Clearly, to test this idea on phytophages, the impact of plant taxonomic or phylogenetic diversity on insect richness would have to be factored into an analysis (see e.g. [26],[28]), which may be difficult to achieve given current data for the region and taxonomic constraints for many insect groups. One alternative approach is to determine whether taxa that are not directly dependent on plant diversity show a pattern of richness in the FB and SKB that represents an outlier in a global analysis, or whether they reflect the general global pattern.

Here, we examine this question by focusing on ants, a well-studied group that is abundant in most ecosystems worldwide [29],[30]. Ants for the most part do not have an evolved close specialist relationship with plants and their biodiversity patterns should thus represent independent data. Exceptions where ant species depend on specific plant species do occur, especially in the tropics, but are rare in the FB and SKB. Ants can use plant resources either for food or for nest sites. While some ants thus use plant-derived foods like seeds and nectar, many ant species also use other food resources like arthropod prey or carcasses and some species are specialist predators, including a not negligible number of those in our study area. Even those species that rely mainly on plant-derived food resources like seed harvesters do typically accept these resources from many different plant species. We thus don't consider relationships like myrmecochory as specialist relationships as they are asymmetric associations such that whilst plants rely on ants for seed dispersal services, ants are not dependent only on these plants [20]. For example in the fynbos indigenous ant species have been observed transporting the seeds of a number of invasive alien plants (see e.g. [31]). Similarly while some ants do use specific plant-provided resources for their nests, many do construct their nests in the soil or can use a variety of different cavities for them. This is especially true in the often litter poor, low canopy habitats of the FB and SKB where many species do nest directly in the soil. Even some of the species that construct their nests above-ground like several species belonging to the genus *Crematogaster* can construct their nests in shrubs belonging to different species and are thus not dependent on a specific plant species.

Ant species richness has been shown to follow the typical latitudinal richness pattern of many taxonomic groups, in the Americas and Europe [32],[33],[34],[35] as well as globally [36]. Positive relationships between ant species richness and either productivity or temperature (often used as a proxy for energy) have likewise been observed at regional to global scales [33],[34],[35],[36],[37],[38] suggesting that the latitudinal pattern is at least in part a consequence of mechanisms related to energy availability. Energy can be expected to be related to rates of local extinction as highly productive sites can support denser populations which would be less likely to go extinct and because more productive sites may have a higher abundance of rare resources. Similarly temperature may also be related to diversity by increasing diversification rates [2],[37],[39]. Temperature or precipitation will also affect foraging time and thus access to resources and in this way also mediate the relationship between productivity and ant diversity [38],[40],[41]. Thus, we compare local richness of ants from sites in the FB and SKB with that from local sites in a range of habitat types worldwide. In particular we examine whether the data from the FB and SKB falls within the general global relationships between ant species richness and two energy-related predictors – normalised difference vegetation index (NDVI, used as a proxy for productive energy) and temperature. Such a comparison has not been made previously, and few studies have sought to interpret measures of local invertebrate diversity in the FB and SKB in a global context, as has been done for plants (see above). In previous investigations, Giliomee [21] and Majer & Greenslade [42] compared ant diversity as well as diversity of some other taxa of the FB with some data from other Mediterranean type habitats in Australia, California, Chile, and Israel, but not with a wider range of habitats and the scale of the comparisons is not always clear. These studies were an important first step but their data on ant richness in the FB is local data from a few sites in only a small part of the biome and thus may not be representative of some other vegetation types within the FB. These studies also do not take the underlying drivers of ant diversity like climate or energy availability into consideration when making comparisons with other regions. In contrast Dunn *et al.* [36] examined climatic drivers of hemispheric asymmetry in ant species richness using a global dataset. However, their Figure 1 shows clearly that their South African data were not collected from the FB and SKB.

**Figure 1:**
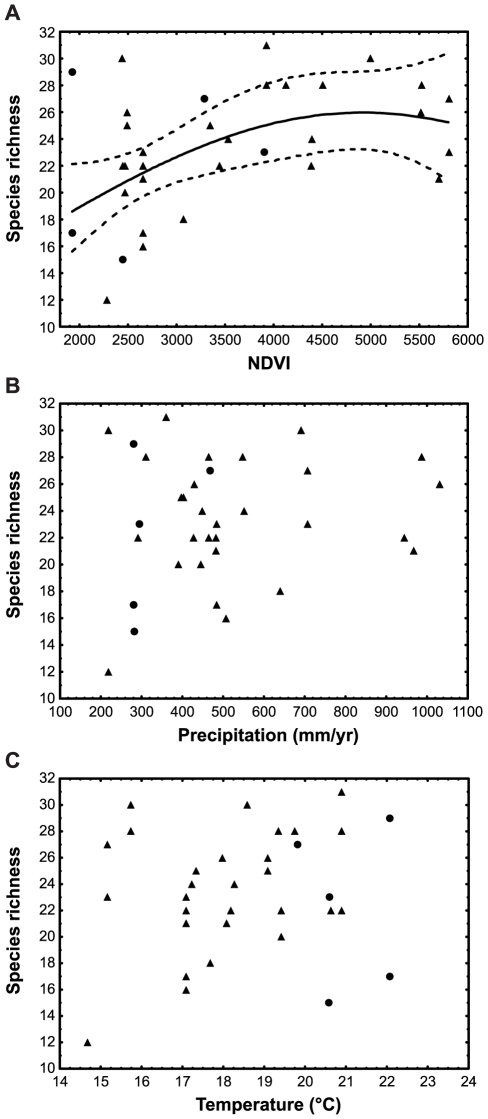
Relationships between ant species richness and energy and climate variables within the FB and SKB. (A) productive energy, (B) mean annual precipitation, (C) mean temperature. Triangles and circles refer to data from FB (*n* = 29) and SKB (*n* = 5) sites respectively. NDVI is taken as a surrogate of productive energy. All measures are averaged over several years to account for natural variability (see **Appendix S3** for methods). Ant species richness was ln(x+1)-transformed for all analyses and regression line and confidence intervals were backtransformed. The regression line and 95% confidence intervals for NDVI are shown. The relationships between ant species richness and precipitation or temperature were not significant. Regression for NDVI: ln(species richness+1) = 2.4325983+0.00035032780 * NDVI−0.000000035595287 * NDVI^2^, *r* ^2^ = 0.22, *F*_2,31_ = 4.50, *P* = 0.0192.

## Results

Within our sites ant species richness did not differ significantly between the FB and the SKB (23.5±4.5 (*n* = 29) *vs.* 22.4±6.3 (SD) (*n* = 5); *t*-test on ln(x+1)-transformed data, *t*_32_ = 0.64, *P* = 0.5277). These figures compare to a mean species richness of 22.8±23.7 ranging from 0 to 177 (*n* = 331) in the global dataset.

Within the FB and SKB the relationship between ant species richness and the normalised difference vegetation index (NDVI, used as a proxy for productivity) was unimodal, peaking at average productivity for FB sites, but above the upper extreme for productivity among the SKB sites in our sample (**Figure 1A**). The only SKB site coming close is an ecotonal site which includes some FB elements. The relationships between species richness and precipitation or temperature were not significant (**Figures 1B and 1C**).

**Figure 2:**
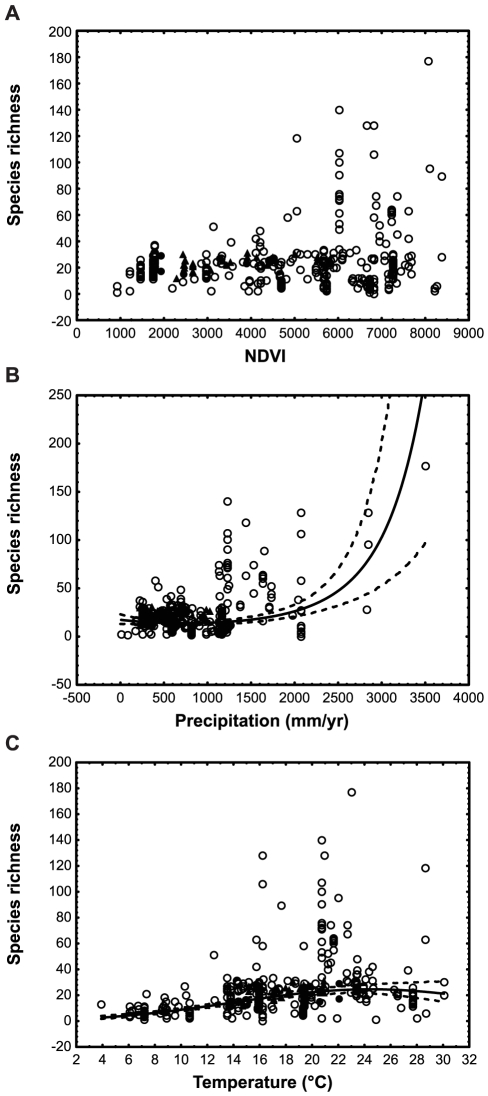
Comparison of ant species richness in the FB and SKB with that of sites worldwide. The relationships between global ant species richness and (A) productive energy, (B) mean annual precipitation, and (C) mean temperature are shown with the data from the FB and SKB superimposed. NDVI is used as a surrogate of productive energy. Clear circles represent global data extracted from the literature (*n* = 331) while triangles and filled circles refer to our own data from FB (*n* = 29) and SKB (*n* = 5) sites respectively. Ant species richness was ln(x+1)-transformed for all analyses. The backtransformed regression lines and 95% confidence intervals for precipitation and temperature are shown. The confidence interval for high values of precipitation is very large and thus not fully shown. The regression for NDVI was not significant for the global dataset. Regressions are: mean annual precipitation: ln(species richness+1) = 2.9100926−0.00056787062 * precipitation+0.00000038199406 * precipitation^2^, *r* ^2^ = 0.86, *F*_2,328_ = 15.51, *P*<0.0001; temperature: ln(species richness+1) = 0.48074860+0.22574050 * temperature−0.0046141963 * temperature^2^, *r* ^2^ = 0.30, *F*_2,328_ = 68.55, *P*<0.0001.

For the global data set, ant species richness followed the expected latitudinal pattern and was highest in the tropics (overall General Linear Model (GLM) including hemisphere and linear and squared terms for latitude as factors; **Table 1**). However, a model including temperature as well as habitat type in addition to hemisphere (**Table 2**) was a much better fit based on Akaike weights [43] than the above model based on latitude. This indicates that the latitudinal pattern may at least in part be due to a latitudinal gradient in temperature. By contrast with the South African analyses, ant species richness at the global scale was not significantly related to NDVI (**Figure 2A**), but showed significant curvilinear relationships with precipitation and temperature (**Figures 2B and 2C**).

**Table 1:** General linear model (type 3) showing the effects of hemisphere and latitude^a^ on ant species richness^b^ (Akaike weight: 6.77 * 10^−13^ c).

Effect	Num df	SSQ	MSQ	Den df	*F*	*P*
Intercept	1	239.754	239.754	326	508.65	<0.0001
Hemisphere	1	4.893	4.893	326	10.38	0.0014
Latitude	1	1.242	1.242	326	2.63	0.1055
Latitude^2^	1	8.325	8.325	326	17.66	<0.0001
Residual	326	153.662	0.471			

a Latitude is the absolute value rounded to the nearest degree.

b Species richness was ln(x+1)-transformed for analysis.

c A global dataset on ant species richness derived from the literature that excludes sites from the FB and SKB is used. Akaike weights are based on a set of all possible models testing effects of hemisphere, latitude (linear and squared terms), habitat type (see **Table 2** for a list of habitat types), temperature (linear and squared terms), and NDVI (linear and squared terms) on ant species richness. This simple model is a much worse fit than the model including habitat type and temperature shown in **Table 2**.

**Table 2:** General linear model (type 3) showing the effects of hemisphere, habitat type^a^, and temperature on ant species richness^b^ (Akaike weight: 0.656241^c^).

Effect	Num df	SSQ	MSQ	Den df	*F*	*P*
Intercept	1	1.041	1.041	319	2.06	0.0403
Hemisphere	1	13.715	13.715	319	34.52	<0.0001
Habitat type	7	30.890	4.413	319	11.11	<0.0001
Temperature	1	16.409	16.409	319	41.30	<0.0001
Temperature^2^	1	8.316	8.316	319	20.93	<0.0001
Residual	319	126.758	0.397			

a Habitat types: arid shrubland, other shrubland (including a variety of shrublands, scrublands and thickets and other bush dominated habitat types), desert, forest, grassland, savanna, wetland, woodland), and temperature on ant species richness.

b Species richness was ln(x+1)-transformed for analysis.

c A global dataset on ant species richness derived from the literature that excludes sites from the FB and SKB is used. The model shown is the best model based on Akaike weights from a set of all possible models testing the above factors and also the effects of linear and squared terms of latitude (absolute value rounded to the nearest degree) and NDVI (productive energy).

When superimposing the data from our sites in the FB and SKB on the global pattern the sites from the two hotspots cluster around the fitted line of the global regression of ant species richness on temperature and fall within the scatter of the global dataset (**Figure 2C**). Indeed when using a Generalized Linear Model with poisson error and log link including linear and squared terms for temperature on the combined dataset we found no significant difference between the species richness of our sites in the FB and SKB and the species richness of the sites elsewhere (*F*_1,361_ = 0.52, *P* = 0.47). The ant richness of these two plant diversity hotspots is thus no higher than expected for a location with that temperature. As we found no significant relationship between ant species richness and NDVI at the global scale, we could not statistically examine whether ant species richness differs between our sites and those in other habitats worldwide when correcting for productivity in the same way as done above correcting for the effect of temperature. However, the data from the FB and SKB fall within the general scatter when superimposing them on the graph plotting ant species richness *vs.* NDVI (**Figure 2A**). In contrast some of the sites in the FB and SKB seem to have relatively high ant species richness given the amount of precipitation they receive though they still fall within the general scatter (**Figure 2B**). Using a GLM with poisson error and log link including linear and squared terms for mean annual precipitation on the combined dataset we found that the species richness of our sites in the FB and SKB is higher than the species richness of the sites in other parts of the world with similar precipitation (*F*_1,361_ = 8.83, *P* = 0.0032; 24.4 species for the sites in the FB or SKB *vs.* 15.3 species for the sites elsewhere based on the back-transformed least squares means from the GLM).

**Figure 3:**
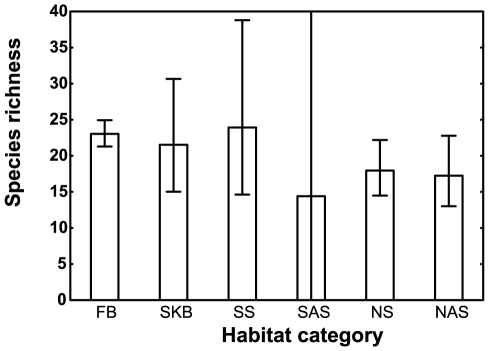
Comparison of ant species richness in the FB and SKB with that of similar habitats. Data for other shrublands and arid shrublands were extracted from the literature and comprise a variety of different vegetation types from desert shrublands to thickets. The other southern hemisphere shrublands and arid shrublands are other South African vegetation types, such as Nama-karoo, and Australian and South American shrublands and arid shrublands. Error bars show 95% confidence intervals. The confidence interval for other southern hemisphere arid shrublands is wide and thus not shown fully, as only the fact that the SKB falls within it is of interest. FB Fynbos biome (*n* = 29), SKB Succulent karoo biome (5), SS other Southern hemisphere shrublands (6), SAS other Southern hemisphere arid shrublands (3), NS Northern hemisphere shrublands (33), NAS Northern hemisphere arid shrublands (16).

Comparing GLMs with the factors hemisphere, habitat type, and linear and squared terms for latitude, NDVI, and temperature shows that habitat type is an important predictor of ant species richness. The best model for ant species richness using the above predictor variables in the global dataset (based on Akaike weights) includes habitat type as a significant factor along with hemisphere and linear and squared terms of temperature but does not include NDVI or latitude (Akaike weight: 0.656241; **Table 2**). For this reason we also separately compared the ant species richness data from the FB and SKB with the data from other regions with a similar vegetation type using a GLM with habitat type as factor. This overall analysis was not significant, providing a further indication that the ant species richness of the FB and SKB is not exceptional (species richness (ln(x+1)-transformed for analysis): *F*_5,86_ = 1.40, *P* = 0.2333; **Figure 3**).

## Discussion

The high regional plant diversity at the southern tip of Africa represents an outlier in the general latitudinal pattern of plant diversity [11],[15],[17]; but a similar pattern is not found in several other taxa (e.g. [28],[44]). The former is a result of high species turnover between sites with many endemic species [17]. By contrast, local plant diversity varies between sub-regions and vegetation types but is similar to other Mediterranean-style ecosystems [17],[18] and is also within the range of other South African biomes [45]. Here, using the first comparison of insect richness in the FB and SKB with that of data from across the globe, we show that the local richness of ants is also not exceptional. Rather the diversity of this group of insects, which for the most part do not have a close specialist relationship with the CFR plants, follows expectations based on diversity-energy and diversity-climate relationships.

These energy relationships are likely underpinned by different mechanisms. Primary productivity (for which NDVI was used as a surrogate) can be expected to affect ant richness in a variety of ways, including by reducing extinction rates and increasing the abundance of rare resources [5]. Temperature as a measure related to ambient energy could affect ant species richness either by increasing diversification rates (e.g. [2],[37],[39]) or by influencing activity time and thus access to resources or foraging efficiency [38],[40],[41]. Our data also indicate that the mechanisms through which energy regulates ant species richness may differ depending on the spatial scale and region (see also [34],[37]), with the global temperature-related pattern locally modified through productivity within the FB and SKB. These findings are in keeping with those of other studies [36],[37].

The global comparison also indicated that whilst local species richness of ants in the FB and SKB is not exceptionally high, it is also not especially low. When repeating the same analyses for generic richness – a rough proxy for functional diversity – we find that results on generic richness are largely similar to those for species richness with the local genus richness of the FB and SKB not being exceptional when compared to the global dataset (results not shown). The exception is that the genus richness of the FB may be higher than that of similar shrublands in the northern hemisphere. However, because genus richness was often not available for sites in the global dataset these analyses have lower sample sizes than those for species richness and should be considered as preliminary only. Overall our results show that earlier statements that insect diversity in the Fynbos Biome is low (e.g. [20],[21]) are certainly incorrect for ants at the local scale, and likely also at broader scales as other recent studies have shown (e.g. [25],[26]).

Regional diversity is a result of both local species richness and turnover, and the Fynbos Biome is known for very high spatial turnover in plant species composition, resulting in high regional diversity [17],[45]. Soil diversity, moisture gradients, topographic heterogeneity and, transient, fire-associated niches are among the environmental factors that are thought to have contributed to high speciation in plants in the FB, while fire is also thought to be instrumental in maintaining the coexistence of species [12],[14], so leading to the average local, but high turnover and regional plant diversity. Of these factors soil diversity [29] can be expected to affect ant diversity patterns as some species prefer specific soil types (see pages 15–16 in [46] for a local example of two related species that occur in our study area and differ in preferring different soil types). Similarly moisture gradients [29],[47] can be expected to affect ant diversity patterns and indeed seemed likely to affect ant diversity in the region based on a significant positive relationship of local ant genus richness with precipitation in our sites (data not shown) even though local ant species richness in our sites tended to be higher than expected based on the global relationship between ant species richness and mean annual precipitation, indicating that other factors did play an important role. By contrast the dissected landscape seems unlikely to have a strong effect on ants, whose young queens can disperse over larger distances than the seeds of the many myrmecochorous plants in the FB, thus making it likely that species turnover in ants is lower than in plants with ant dispersed seeds. Similarly, subterranean nests may make many ant species resilient to fires [48] - exclusively arboreal ant species are rare in the mostly low canopies of the FB and SKB and many of the vegetation types in the two biomes have relative little litter for use as nesting substrate. In consequence, it might be expected that regional diversity and turnover in ants in the FB and SKB might not be as high as it is for plants. Although we focussed on the local scale here, an initial species count across all of our sites (151 species overall with 133 species in the FB and 62 species in the SKB) and consideration of the species accumulation curves for our sites in the FB and SKB [49],[50] suggests that by contrast with the vascular plants it is also unlikely that the FB or SKB have an exceptional regional ant richness, and they certainly do not have a regional ant species richness as high as that reported for tropical rainforests, where much higher species richness has been reported, often from smaller scale studies. For example, estimates of regional richness vary from 18 in an arid system in Israel [51] to 73 in Western Australia [42], to 160 in a South African savanna [52], to 437 in a Costa Rican lowland rainforest [53] (**Appendix S7**).

The FB and SKB are not only known for their high regional plant species richness and high turnover in plant species but also for high levels of endemism. The location of the two biomes at the tip of a continent with adjacent biomes that experience different climatic conditions makes it more likely that species found in the area will be restricted to it. Indeed Robertson [54] found 90.5% of ant species (excluding not yet described species) found in the Cape Peninsula – which is located at the heart of the FB – had localised distributions, i.e. they only were known to occur in South Africa itself or in South Africa and one additional country. This contrasted with only 16.9% similarly localised species in Mkomazi Game Reserve in Tanzania where many species that are widespread in savannahs or forests were found. However, ant species whose distribution is restricted to the CFR and maybe some adjacent biomes do not need to have a restricted distribution within the FB and the relationship between this regional endemism and turnover among sites within the region is thus not necessarily strong.

Earlier work suggested that some groups of invertebrates may have high species richness in the Fynbos and Succulent Karoo biomes even if this is not true for all groups [19],[21],[22],[23],[24]. Our study focused on a taxon with no close specialist relationship with plants and similar results may be expected for other such taxa. Indeed where insect taxa have previously been found to be extraordinarily diverse in the FB and SKB, these are taxa usually closely associated with plants [22],[23],[24],[25],[27]. Our findings are in line with those from studies searching for indicator taxa which showed that diversity gradients of one taxon are not always a good predictor for those in other taxa; something that is likely more often the case when these groups are not having close specialist relationships with each other and where taxa are also affected by some environmental factors in different ways as is likely the case for the ants and plants in our study area.

## Methods

### Study area

Ants were sampled in the Western Cape Province of South Africa which encompasses the majority of the Fynbos Biome and the Succulent Karoo Biome. Most of the FB is characterized by frequent fires and relatively low productivity shrublands. Rainfall is limiting almost throughout the entire area, but varies substantially depending on exposure to prevailing winds as well as between the strictly winter rainfall area in the west, and the east which also receives substantial summer precipitation [9],[17]. While fynbos vegetation is typical for nutrient-poor soils, areas with higher productivity soils are often covered by the related renosterveld vegetation or by denser thickets [9]. Strandveld occurs in some coastal areas. This vegetation is less fire-prone than fynbos. Forest patches are localized, small and patchily distributed in the region [9] and forest was therefore excluded from this study.

The little rain that falls in the SKB is mostly restricted to the winter. Despite the arid conditions, the SKB has exceptionally high plant diversity and high endemism of plants [9],[10]. SKB vegetation is found on the plains rather than the mountains which are dominated by fynbos vegetation, and on more eutrophic soils. Floristically the SKB is related to both the FB and the adjacent Nama-Karoo [9].

### Ant collection in the FB and SKB

We use data from two ongoing ant monitoring programs in the Western Cape [49],[50]. These programs cover a large part of the extent of both the FB and SKB including the core zone of the FB and can thus be considered representative of them even though our sample size for the SKB is small (see **Appendix S4** for information on study site locations and vegetation types). In this respect this study differs from earlier studies of ants in the FB and SKB that typically were local and didn't encompass the variety of vegetation types in these biomes. This is especially true for the SKB, where even less is known about the insect diversity than for the FB. Our sites were selected to represent the range of environmental conditions available in the area, ranging in altitude from coastal to the summit of one of the highest mountains in the study area (1926 m a.s.l., **Appendix S4**) and including habitats with a range of precipitation patterns and soil types. Overall, we examine 29 FB sites including sites in coastal and mountain fynbos, renosterveld, and strandveld and five SKB sites with different plant species compositions and substrates. One of the FB and one of the SKB sites were ecotonal, comprising elements of the other biome.

Ants were collected twice per year using pitfall traps in October/early November (spring) and late February/March (autumn) when ants are most active in this region [49]. Traps were exposed for five days on each occasion. We use pooled data from the first three sampling events from each study for the current investigation. As the two studies started in separate years this means 16 FB sites and 1 SKB site were sampled from October 2002 to October 2003, while the other 13 FB and 4 SKB sites were sampled from March 2006 to March 2007. Pitfall traps were set in two grids of 2×5 traps per site with a distance of 10 m between traps and a distance of 150–250 m between grids depending on local circumstances. Data from the two grids per site were pooled for analysis. In one program two further grids were sampled per site [49]. These were not included in the present study in order not to bias comparisons between the FB and SKB. Traps were plastic cups of 7 cm diameter partially filled with 50% propylene glycol solution which is non-attractive for ants.

In a first step ants were identified to genus level and then sorted into morphospecies based on taxonomic criteria within these genera. These morphospecies could then be identified to species in many cases. In cases where this was not possible codes like *Pheidole* sp. 4 or *Monomorium* sp. 5 were assigned. The available taxonomic literature does not at present allow naming all of our morphospecies as many ant species in the region still have not yet been described and because for many genera no modern taxonomic revisions are available [54]. Even where modern keys are available, they are sometimes based on limited material and thus may not reflect the full intra-specific variation. For the purpose of this paper the morphospecies are regarded as equivalent to species. A species list with abundances for the different sites is given as **Table S1**. Voucher specimens of all species have been lodged with Iziko South African Museums in Cape Town.

Most sites were located in protected areas. Some sites with mostly natural vegetation that have been disturbed by humans in a variety of ways were included. However, the ant species richness and composition did not differ significantly between these sites and the pristine sites [50]. One of the SKB sites was only included later in the project and only sampled twice instead of three times. Additionally, two sites in the neighbouring Nama-Karoo biome were sampled in the same way and included in our sample of sites from outside the FB and SKB (**Appendix S4**).

### Ant species richness elsewhere

Data on local richness of ants in a range of ecosystems worldwide were extracted from the literature. Species-energy relationships may depend on the grain, extent and period over which samples are taken [5],[55]. Furthermore, number of samples taken, collection method, experience of the collector, and available taxonomic knowledge for the area examined will affect the number of species reported [36],[56]. We aimed to minimize the effect of variation contributed by sampling design by only using data from studies that met predefined criteria similar to those applied by Dunn *et al.* [36] (see details on study selection criteria in **Appendix S1**). We included studies targeting ground-dwelling ants using methods likely to yield a random and relatively comprehensive sample of the ant community at a site. Depending on habitat type and aim of the study different methods were employed by the collectors. For example litter extraction can only be used in sites with reasonable amounts of not too dry litter (e.g. forest habitats) and is thus a frequently employed method in some productive habitats. In contrast pitfall traps are ideal for ant surveys in open habitats like the ones in the FB and SKB, though they are less ideal for very rocky habitats. In order to include data from a range of habitat types covering a wide range of productivity and temperature it was thus necessary to include data from studies using different collection methods. Our own method - pitfall traps - was by far the most commonly used method in the studies included in the global dataset (257 sites) followed by litter extraction (105 sites) and in some of these sites both methods were used, sometimes in combination with additional methods. Alone or in combination these two methods were used in 302 out of 331 sites included in the datasets. However, in order to represent a wider range of habitat types, geographical areas, and productivity and climatic conditions, a few studies using different methods (typically multiple methods) were included in the dataset (see **Appendix S1**). The different sampling protocols employed certainly add to the scatter in the global dataset. However, differences between different methods may not always be as great as sometimes feared. Different methods focused on surface-foraging ants can yield species lists, whose differences in part are caused by collecting rare species with only one method that might just as likely have been picked up with another method [57]. Indeed Ellison et al. [58] found that when comparing species lists from a forest from several methods most were not statistically distinct from each other (this included the comparison between litter samples and pitfall traps). Still a more standardized approach to ant surveys would be highly welcome and facilitate comparisons among ant assemblages in different habitat types and geographical areas. Standardized hand collecting has recently been promoted as a possible way forward [57] while protocols involving multiple methods have also been suggested [59].

Only data from natural and semi-natural sites were included in the global ant richness database. To reduce any bias associated with sampling design like collection methodology or sampling size we used data on species richness from a large sample of sites (331 data points representing 53 separate studies in Africa, the Americas, Australia, Europe, and the Indotropics; not including our FB and SKB sites; see **Appendix S2** for a list of studies from which data were extracted). Six of the 53 studies only reported mean species richness for replicates of the same type. These means were entered in the database as if they represented a single site. Eleven out of the 331 values entered were derived from such means while the others represent single sites.

Largely because it was common to most studies, observed species richness (Sobs) per site was used as an estimator of true species richness, acknowledging that Sobs is typically somewhat lower than actual species richness [60], but also bearing in mind the small effect of sample size found by Dunn *et al.* [36] in their global study. Where necessary, data were extracted from graphs or appendices. In three studies which represented 8.2% of sites included in the analyses, observed species richness was not available and Chao2 estimates were accepted instead. These sites were spread over a range of habitat types. Chao2 is usually higher than observed species richness but comparing them for one of our monitoring projects in the FB and SKB indicates that the difference is often small (16.7%±14.4% SD).

### Energy availability

The latitudinal pattern in species richness observed in many groups - including ants - has often been attributed to energy availability. In a first step we examined whether a latitudinal pattern was present in our global dataset using general linear models (GLM) (type 3) with linear and squared terms of latitude (measured in degrees as absolute values) and hemisphere (North, South) as a fixed factor. Hemisphere was added as ant species richness has previously been shown to differ between hemispheres [36]. In further steps we then examined whether ant species richness is related to energy.

When studying species richness-energy relationships it is important to select a measure of energy availability that is relevant to the taxon concerned [5]. Energy is available to consumer taxa in the form of chemical energy retained in the biomass produced by plants [61] and plant productivity is thus considered an appropriate measure. We used the normalised difference vegetation index NDVI averaged over several years (see **Appendix S3** for details) because it correlates strongly with net primary productivity and is ultimately a result of water availability, temperature, and solar irradiation [5]. Ants are in the main not direct consumers of primary productivity but indirectly depend on it through preying on herbivorous insects. However, many ant species also consume plant material such as nectar, seeds, or elaiosomes attached to seeds, and for some species these are the major food source [29]. In the FB, myrmecochory by ants is widespread [20], while seed harvesters are also frequent in the SKB, and consequently the local ant community can be considered to be more directly dependent on the vegetation than is the case in many regions worldwide. However, seed-dispersing ants take seeds from many plant species and the relationship is thus not highly specific.

Ant diversity has also been shown to be strongly related to temperature [33],[38]. Temperature is not an energy variable itself but determines the use of available energy [38],[40],[41]. Mean annual temperature for several years was used for analyses (see **Appendix S3** for details).

Diversity of many taxa has also been shown to be related to variation in rainfall. Precipitation can be a limiting factor for ant species richness in some semi-arid regions [62],[63] though ant diversity and precipitation are not always correlated (e.g. [35]). Rainfall can have an indirect effect on consumer taxa through effects on plant species richness and productivity and can thus be used as a proxy for productivity. For example colony productivity in the South African ant species *Ocymyrmex foreli* was positively related with mean monthly rainfall [64]. For our dataset mean annual precipitation was strongly correlated with NDVI (**Appendix S6**) and we thus elected not to use precipitation as an independent variable together with NDVI. However, because precipitation might have effects on ant diversity that are unrelated to plant productivity, in a separate set of analyses we examined the relationships between mean annual precipitation and ant richness (see **Appendix S3** for source details).

### Statistical methods

Species richness was ln(x+1)-transformed for all analyses to conform to conditions of normal distribution and to make the variances independent of the mean. In a first step we examined whether the global dataset showed spatial autocorrelation by computing semivariograms (**Appendix S5**). As spatial autocorrelation was found to be negligible in the global dataset we did not include it in further models.

Species richness was compared between the FB and SKB using *t*-tests after first testing for equality of variance (Folded *F* statistic). We then examined how ant richness is affected by energy availability within the FB and SKB. The close spatial and floristic association makes it reasonable to treat the two hotspots together. We used linear and quadratic regression analyses to examine whether ant species richness in the two hotspots is related to NDVI, precipitation, or temperature. In cases where both the linear and the quadratic model are significant the model with the higher adjusted *r* ^2^ is presented. We then repeated this approach for the global dataset. In a next step we superimposed the data of our sites from the FB and SKB onto these global relationships. Where no significant global relationship was found we visually assessed the position of the data from the FB and SKB with regard to the other data. Where the global relationship was significant we did run a Generalised Linear Model (GLM) using the combined dataset. Those models included linear and squared terms of the energy or climate variable and location of the site (our sites in the FB and SKB *vs.* sites elsewhere) as a fixed factor and thus tested whether the ant species richness in the two hotspots differed from other sites with a similar climate or similar energy availability.

Because preliminary analyses using GLM (type 3) showed that ant richness in the global dataset differed among habitat types (arid shrubland, other shrubland (including a variety of shrublands, scrublands and thickets and other bush dominated habitat types), desert, forest, grassland, savanna, wetland, woodland) we also compared the FB and SKB solely with other shrublands and arid shrublands. We first compared the habitat groups using a GLM (type 3) with habitat group as factor (FB, SKB, other shrublands in the Southern hemisphere, other arid shrublands in the Southern hemisphere, other shrublands in the Northern hemisphere, and other arid shrublands in the Northern hemisphere). Habitat categories were grouped by hemisphere as this factor was included in the best fit overall model for ant species richness from the sites outside the FB and SKB. Posthoc tests were conducted to compare our sites from the FB and SKB with the other groups (Tukey-Kramer adjusted for unequal sample sizes). Sample sizes for some groups are small. However, the analyses allow pointing out trends.

Statistical analyses were conducted using IBM SPSS Statistics 19 (SPSS Inc., Chicago, USA, 2010), Statistica version 10 (StatSoft Inc., Tulsa, USA, 2011), and SAS 9.1.3 (SAS Institute Inc., Cary, USA, 2002–2003). Semivariograms were computed using GS+™ Geostatistics for the Environmental Sciences Version 5.1.1 (Gamma Design, Plainwell, USA, 1989–2001).

## Supporting Information

Table S1 **Ant species list and abundances in study sites for FB and SKB.**
(XLS)

Appendix S1 **Detailed methods used to generate database on non-FB and non-SKB ant richness.**
(DOC)

Appendix S2 **List of studies from which data were extracted.**
(DOC)

Appendix S3 **Detailed methods to generate means for energy variables.**
(DOC)

Appendix S4 **Information on study sites within the FB and SKB.**
(DOC)

Appendix S5 **Semivariograms.**
(DOC)

Appendix S6 **Correlation matrices for energy variables and latitude.**
(DOC)

Appendix S7 **Regional ant species richness.**
(DOC)
