# Supplementary material for: The Fynbos and Succulent Karoo Biomes Do Not Have Exceptional Local Ant Richness
Source: PLoS One. 2012 Mar 2;7(3):e31463. doi: 10.1371/journal.pone.0031463 (PMC3292543; doi:10.1371/journal.pone.0031463)
Supplement: Appendix S1 — Detailed methods used to generate database on non-FB and non-SKB ant richness. (DOC) [file pone.0031463.s002.doc]

**Appendix S1. Detailed methods used to generate database on non-FB and non-SKB ant richness**

**Supporting Information for:** B. Braschler, S.L. Chown, and K.J. Gaston: The Fynbos and Succulent Karoo Biomes do not have Exceptional Local Ant Richness

*Retrieval of source studies*

Possible source studies were identified using the first author’s personal collection of papers and by searching Thompson’s ISI Web of Science™ database <http://isiwebofknowledge.com> and the internet (using Google™ <www.google.com>) using the search term “ant species richness” and search terms relating to ecosystems and geographical areas that were underrepresented in the first collection. The internet was included in the search as studies reporting detailed information about species richness of sites are sometimes published in local journals and thus would be missed if only ISI listed journals were searched.

*Criteria used for deciding whether to include sites in the database*

Studies were included in the database for analysis if:

- They targeted ground-dwelling ants (not arboreal ants). This is because many studies only target either ground-dwelling ants or arboreal ants. Excluding arboreal ants made the present analyses conservative as arboreal ant species are most abundant in tropical forests while the low canopy of the CFR shrublands harbours few exclusively arboreal species.
- They used methods aimed at obtaining a broad inventory of the site studied without taxonomic or trophic restrictions. This means that techniques like non-attractive pitfall traps, complete nest surveys through excavation, and litter extraction were accepted. However in most cases bait only studies were excluded as they could reasonably be expected to attract only some species (e.g. seed harvesters in the case of seed baits). Studies that complemented baiting techniques with other techniques like pitfall trapping or intensive hand collecting were included as was one study that used a variety of baits. The vast majority of studies (302 out of 331 sites) included used either pitfall traps (257 sites) or litter extraction (105 sites) including some studies that used both collection methods (sometimes in combination with other methods). In order to represent a wider range of habitat types, geographical areas, and productivity and climatic conditions, a few studies using different methods (typically multiple methods) were included in the dataset. This includes quadrate sampling, baiting, hand collections, excavation, and soil samples, as well as a few instances where methods that may also trap arboreal ants were included in multi-method sampling protocols together with some of the already listed methods (sweep netting, Malaise trap, yellow pan traps, and beating).
- The study had a reasonable intensity to obtain a general impression of the magnitude of ant species richness of the site. This excluded studies with very few samples per site.
- The grain at which samples were taken was roughly comparable to that of our CFR sites.
- They reported point species richness either for the separate sites (plots, replicates) or mean point species richness for such sites. If means were reported then they were entered into the analyses as one sample. Sites were only considered as independent points if they were far enough from each other to be reasonably expected not to sample the same community. This led to the exclusion of some small scale experimental studies and to only partial inclusion of some other studies.
- The plots did not cover more than one ecosystem (e.g. ecotonal transects going from forest into the adjoining grassland were excluded). If means were reported they were only included in our analyses if they were not derived from sites in several different ecosystems.
- They were done in natural or semi-natural habitat that was not subjected to an experimental treatment (e.g. control plots of studies). This rule had to be somewhat flexible so as not to omit whole habitat types for intensively used areas like Western Europe. Studies reporting ant species richness for managed grasslands and forests were thus included, though intensive agriculture or monoculture pine plantations were not.
- They reported the location of the sites and the vegetation type. Whenever possible coordinates reported in the study were used for location. In some cases alternative ways to obtain the location had to be used; e.g. estimates based on maps reported in the paper, information from related papers, or reference to websites of a research station. Accuracy of the reported location varied between studies. However, using a larger grain size (0.15º grid cells) for energy variables did not yield different patterns from the finer grain used in the paper.
- If more than one study with the same authors was from the same area it was assumed that the sites are the same and only one study was included in our analyses.
- Sites within the FB and SKB were not included in our database, though sites in other South African biomes from papers that also studied sites in the FB or SKB were included in the database.

Morphospecies were considered equivalent to species for the calculation of species richness. Most data included in our dataset were collected during the last two decades. However, one value dates back to 1973. It was included as it represented a site with extremely low productivity thus extending the range of NDVI covered in our analysis. A list of studies from which data were included in the final database is given in **Appendix S2** in Supporting Information.
