# Supplementary material for: The Fynbos and Succulent Karoo Biomes Do Not Have Exceptional Local Ant Richness
Source: PLoS One. 2012 Mar 2;7(3):e31463. doi: 10.1371/journal.pone.0031463 (PMC3292543; doi:10.1371/journal.pone.0031463)
Supplement: Appendix S2 — List of studies from which data were extracted. (DOC) [file pone.0031463.s003.doc]

**Appendix S2. Studies from which data were extracted**

**Supporting Information for:** B. Braschler, S.L. Chown, and K.J. Gaston: The Fynbos and Succulent Karoo Biomes do not have Exceptional Local Ant Richness

Andersen AN (1992) Regulation of "momentary" diversity by dominant species in exceptionally rich ant communities of the Australian seasonal tropics. Am Nat 140: 401-420.

Andersen AN (1997) Functional groups and patterns of organization in North American ant communities: a comparison with Australia. J Biogeogr 24: 433-460

Andersen AN, Hoffmann BD, Müller WJ, Griffiths AD (2002) Using ants as bioindicators in land management: simplifying assessment of ant community responses. J Appl Ecol 39: 8-17.

Andersen AN, Hoffmann BD, Somes J (2003) Ants as indicators of minesite restoration: community recovery at one of eight rehabilitation sites in central Queensland. Ecol Manage Restor 4: S12-S19.

Arnan X, Rodrigo A, Retana J (2006) Post-fire recovery of Mediterranean ground ant communities follows vegetation and dryness gradients. J Biogeogr 33: 1246-1258.

Barrow L, Parr CL, Kohen JL (2007) Habitat type influences fire resilience of ant assemblages in the semi-arid tropics of Northern Australia. J Arid Environ 69: 80-95.

Bestelmeyer BT, Schooley RL (1999) The ants of the southern Sonoran desert: community structure and the role of trees. Biodivers Conserv 8: 643-657.

Bestelmeyer BT, Wiens JA (2001) Ant biodiversity in semiarid landscape mosaics: The consequences of grazing vs. natural heterogeneity. Ecol Appl 11: 1123–1140.

Bestelmeyer BT, Wiens JA (2001) Local and regional-scale responses of ant diversity to a semiarid biome transition. Ecography 24: 381–392.

Bolger DT (2007) Spatial and temporal variation in the Argentine ant edge effect: Implications for the mechanism of edge limitation. Biolog Conserv 136: 295-305.

Braschler B (2003) Ameisen (Hymenoptera: Formicidae). Fauna und Flora auf dem Eisenbahngelände im Norden Basels (ed. by Burckhardt D, Baur B, Studer A), pp. 110-114. Monogr Entomol Ges Basel 1. Entomologische Gesellschaft Basel, Basel.

Braschler B, Baur B (2005) Experimental small-scale grassland fragmentation alters competitive interactions among ant species. Oecologia 143: 291–300.

Brühl CA, Mohamed M, Linsenmayr KE (1999) Altitudinal distribution of leaf litter ants along a transect in primary forests on Mount Kinabalu, Sabah, Malaysia. J Trop Ecol 15: 265-277.

Campos RBF, Schoereder JH, Sperber CF (2007) Small-scale patch dynamics after disturbance in litter ant communities. Basic Appl Ecol 8: 36-43.

Catalina Estrada M, Fernando Fernández C (1999) Diversidad de hormigas (Hymenoptera: Formicidae) en un gradiente sucesional del bosque nublado (Nariño, Colombia). Rev Biolog Trop 47: no.1-2.

Dauber J, Wolters V (2005) Colonization of temperate grassland by ants. Basic Appl Ecol 6: 8-91.

Dauber J, Hirsch M, Simmering D, Waldhardt R, Otte A, Wolters V (2003) Landscape structure as an indicator of biodiversity: matrix effects on species richness. Agric Ecosyst Environ 98: 321–329.

Deblauwe I, Dekoninck W (2007) Diversity and distribution of ground-dwelling ants in a lowland rainforest in southeast Cameroon. Insect Soc 54: 334–342.

Debuse VJ, King J, House APN (2007) Effect of fragmentation, habitat loss and within-patch habitat characteristics on ant assemblages in semi-arid woodlands of eastern Australia. Landscape Ecol 22: 731–745.

Ellison AM, Farnsworth EJ, Gotelli NJ (2002) Ant diversity in pitcher-plant bogs of Massachusetts. Northeast Nat 9: 267–284.

Ellison AM, Chen J, Díaz D, Kammerer-Burnham C, Lau M (2005) Changes in ant community structure and composition associated with hemlock decline in New England. Proceedings of the 3rd Symposium on Hemlock Woolly Adelgid in the Eastern United States (ed. by Onken B, Reardon R), pp. 280-289. US Department of Agriculture - US Forest Service - Forest Health Technology Enterprise Team, Morgantown.

Fabricius C, Burger M, Hockey PAR (2003) Comparing biodiversity between protected areas and adjacent rangeland in xeric succulent thicket, South Africa: arthropods and reptiles. J Appl Ecol 40: 392–403.

Fisher BL Robertson HG (2002) Comparison and Origin of Forest and Grassland Ant Assemblages in the High Plateau of Madagascar (Hymenoptera: Formicidae). Biotropica 34: 155–167.

Gómez C, Pons P, Bas JM (2003) Effects of Argentine ant Linepithema humile on seed dispersal and seedling emergence of Rhamnus alaternus. Ecography 26: 532-538.

Gotelli NJ, Ellison AM (2002) Assembly rules for New England ant assemblages. Oikos 99: 591-599.

Heatwole H (1996) Ant assemblages at their dry limits: the northern Atacama Desert, Peru, and the Chott El Djerid, Tunisia. J Arid Environ 33: 449-456.

Herbers JM (1985) Seasonal structuring of a north temperate ant community. Insect Soc 32: 224-240.

Herbers JM (1989) Community structure in north temperate ants: temporal and spatial variation. Oecologia 81: 201-211.

Holway DA (1998) Factors governing rate of invasion: a natural experiment using Argentine ants. Oecologia 115: 206-212.

Holway DA (1998) Effect of Argentine ant invasions on ground-dwelling arthropods in northern California riparian woodlands. Oecologia 116: 252-258.

Kaspari M, O’Donnell S, Kercher JR (2000) Energy, density, and constraints to species richness: Ant assemblages along a productivity gradient. Am Nat 155: 280-293.

Lassau SA, Hochuli DF (2004) Effects of habitat complexity on ant assemblages. Ecography 27: 157-164.

Lobry de Bruyn LA (1993) Ant composition and activity in naturally-vegetated and farmland environments on contrasting soils at Kellerberrin, Western Australia. Soil Biol Biochem 25: 1043-1056.

Majer JD (1992) Ant recolonisation of rehabilitated bauxite mines of Pocos de Caldas, Brazil. J Trop Ecol 8: 97-108

Majer JD, Delabie JHC (1999) Impact of tree isolation on arboreal and ground ant communities in cleared pasture in the Atlantic rain forest region of Bahia, Brazil. Insect Soc 46: 281–290.

Oliver I, Beattie AJ (1996) Designing a cost-effective invertebrate survey: a test of methods for rapid assessment of biodiversity. Ecol Appl 6: 594-607.

Ottonetti L, Tucci L, Santini G (2006) Recolonization patterns of ants in a rehabilitated lignite mine in central Italy: Potential for the use of Mediterranean ants as indicators of restoration processes. Restor Ecol 14: 60–66.

Palladini JD, Jones MG, Sanders NJ, Jules ES (2007) The recovery of ant communities in regenerating temperate conifer forests. Forest Ecol Manag 242: 619–624.

Parr CL, Bond WJ, Robertson HG (2002) A preliminary study of the effect of fire on ants (Formicidae) in a South African savanna. Afr Entomol: 10, 101–111.

Parr CL, Robertson HG, Biggs HC, Chown SL (2004) Response of African savanna ants to long-term fire regimes. J Appl Ecol 41: 630–642.

Ratchford JS, Wittman SE, Jules ES, Ellison AM, Gotelli NJ Sanders NJ (2005) The effects of fire, local environment and time on ant assemblages in fens and forests. Divers Distrib 11: 487–497.

Retana J, Cerdá X (2000) Patterns of diversity and composition of Mediterranean ground ant communities tracking spatial and temporal variability in the thermal environment. Oecologia 123: 436-444.

Ríos-Casanova L, Valiente-Banuet A, Rico-Gray V (2006) Ant diversity and its relationship with vegetation and soil factors in an alluvial fan of the Tehuacán Valley, Mexico. Acta Oecol 29: 316-332.

Robertson HG (2002) Comparison of leaf litter ant communities in woodlands, lowland forests and montane forests of north-eastern Tanzania. Biodivers Conserv 11: 1637–1652.

Robertson HG, van Noort S (2003) Effects of agricultural practices on ants and other Hymenoptera in four farming areas of South Africa. GEF Conservation Farming Project Report. Iziko museums of Cape Town.

Rojas P, Fragoso C (2000) Composition, diversity, and distribution of a Chihuahuan Desert ant community (Mapimí, México). J Arid Environ 44: 213–227.

Silva RR, Machado Feitosa RS, Eberhardt F (2007) Reduced ant diversity along a habitat regeneration gradient in the southern Brazilian Atlantic Forest. Forest Ecol Manag 240: 61–69.

Soares SM, Schoereder JH, DeSouza O (2001) Processes involved in species saturation of ground-dwelling ant communities (Hymenoptera, Formicidae). Austral Ecol 26: 187-192.

Sobrinho TG, Schoereder JH (2007) Edge and shape effects on ant (Hymenoptera: Formicidae) species richness and composition in forest fragments. Biodivers Conserv 16: 1459–1470.

Theunis L, Gilbert M, Roisin Y, Leponce M (2005) Spatial structure of litter-dwelling ant distribution in a subtropical dry forest. Insect Soc 52: 366–377.

Torres JA (1984) Niches and Coexistence of Ant Communities in Puerto Rico: Repeated Patterns. Biotropica 16: 284-295.

Watt AD, Stork NE, Bolton B (2002) The diversity and abundance of ants in relation to forest disturbance and plantation establishment in southern Cameroon. J Appl Ecol 39: 18–30.
