# Supplementary material for: The Fynbos and Succulent Karoo Biomes Do Not Have Exceptional Local Ant Richness
Source: PLoS One. 2012 Mar 2;7(3):e31463. doi: 10.1371/journal.pone.0031463 (PMC3292543; doi:10.1371/journal.pone.0031463)
Supplement: Appendix S3 — Detailed methods to generate means for energy variables. (DOC) [file pone.0031463.s004.doc]

**Appendix S3. Detailed methods to generate means for energy variables**

**Supporting Information for:** B. Braschler, S.L. Chown, and K.J. Gaston: The Fynbos and Succulent Karoo Biomes do not have Exceptional Local Ant Richness

*Normalised difference vegetation index NDVI*

NDVI values were derived from MODIS/Terra Vegetation Indices Monthly L3 Global 0.05Deg CMG V005 < https://lpdaac.usgs.gov/lpdaac/products/modis_product_table/vegetation_indices/monthly_l3_global_0_05deg_cmg/v5/terra >. We calculated mean yearly NDVI in a two step procedure. First, we averaged the NDVI values for each calendar month separately over several years (February 2000 – August 2005 and January – December 2007). In the second step these means for the twelve calendar months were then averaged to get a mean yearly value. By averaging first the values for each calendar month over the different years we avoided biased yearly means due to missing monthly values in the dataset. We averaged NDVI values over several years to allow for among year variations. The period chosen included years during which our own data were collected and a period leading up to that date. The NDVI values were obtained for 0.05º grid cells. In some instances using mean values over a larger area may lead to the NDVI value being unrepresentative of the productivity of the study site itself. However, the size of the grid cells reflects the fact that as generalist herbivores, predators and scavengers ants may indirectly draw resources from a larger area than just their immediate surroundings. Furthermore, some studies included in our database did not report the location of their sites at a fine grain and thus using a finer resolution would have provided artificial precision. For our own study sites in the FB and SKB, NDVI values were correlated with directly measured mean vegetation height (*n* = 34, *r*_s_ = 0.42, *P* = 0.014).

*Temperature*

Mean temperature values were obtained from the dataset MODIS/Terra Land Surface Temperature/Emissivity Monthly L3 Global 0.05Deg CMG < https://lpdaac.usgs.gov/lpdaac/products/modis_products_table/land_surface_temperature_emissivity/monthly_l3_global_0_05deg_cmg/mod11c3 >. The dataset contained monthly temperatures for day and night for 0.05º grid cells for March 2000 - December 2006. We averaged day and night temperature to obtain mean temperatures before calculating means following the two-step procedure outlined for NDVI.

*Precipitation*

Mean annual precipitation was derived from the WorldClim database (Hijmans *et al.*, 2005).

Hijmans RJ, Cameron SE, Parra JL, Jones PG, Jarvis A (2005) Very high resolution interpolated climate surfaces for global land areas. Internat J Climat 25: 1965-1978.
