# Supplementary material for: The Fynbos and Succulent Karoo Biomes Do Not Have Exceptional Local Ant Richness
Source: PLoS One. 2012 Mar 2;7(3):e31463. doi: 10.1371/journal.pone.0031463 (PMC3292543; doi:10.1371/journal.pone.0031463)
Supplement: Appendix S4 — Information on study sites within the FB and SKB. (DOC) [file pone.0031463.s005.doc]

**Appendix S4. Information on study sites**

**Supporting Information for:** B. Braschler, S.L. Chown, and K.J. Gaston: The Fynbos and Succulent Karoo Biomes do not have Exceptional Local Ant Richness

Abbreviations: FB Fynbos Biome, SKB Succulent Karoo Biome

**Figure S1. Locations of study sites in the FB and SKB.** Study sites were spread across the Western Cape Province in South Africa in order to represent different vegetation types, soil types, and climatic conditions within the FB and SKB. Where sites were selected close to each other they represent different habitat conditions or vegetation types, e.g. rocky mountain slope *vs*. sandy plain. Precipitation gradients run from the wetter coastal areas across the mountains to the drier inland plateau and from the strictly winter rainfall area in the west towards the east which also can receive substantial summer precipitation. The dense cluster of sites in the north-west is the two-dimensional representation of sites located at different altitudes and aspects in the Cederberg Mountains with the highest elevation site at 1926 m on the summit of Sneeukop, one of the highest mountains in the province. Altitude for the sites is in **Table S2** in this appendix. Labels refer to panels in **Figure S2** in this appendix which show example photographs of different vegetation units. Two additional sites located within the Nama-Karoo Biome which covers the north-east of the Western Cape Province were sampled with the same methods and included in the global dataset. In each site two grids with ten pitfall traps were set and sampled three times over the period of one and a half years.

**Figure S2.**

**A) Lamberts Bay: Lambert’s Bay Strandveld and Cape Seashore Vegetation**

**
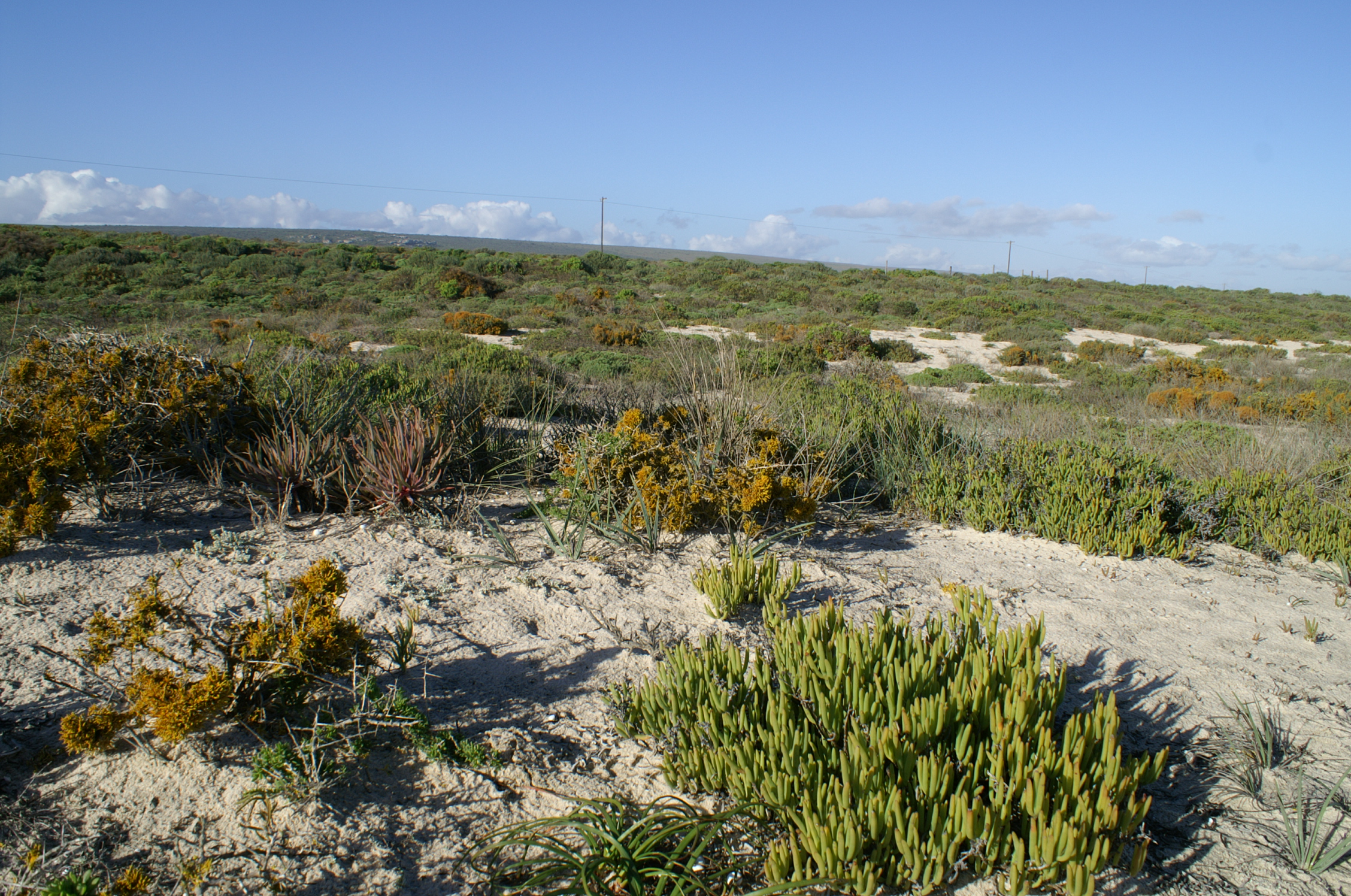

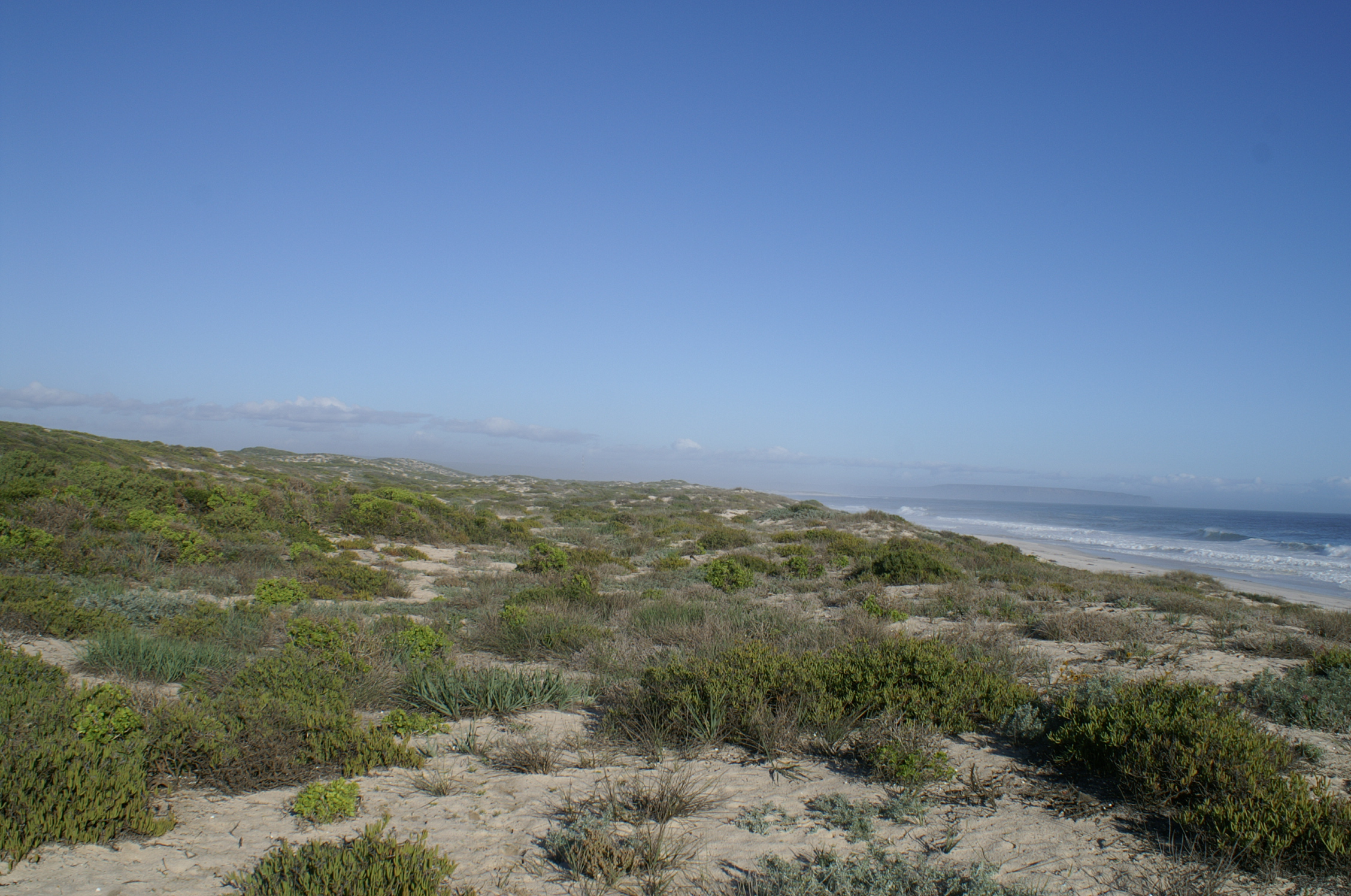
**

**B) Somerset West: Cape Winelands Shale Fynbos**

**
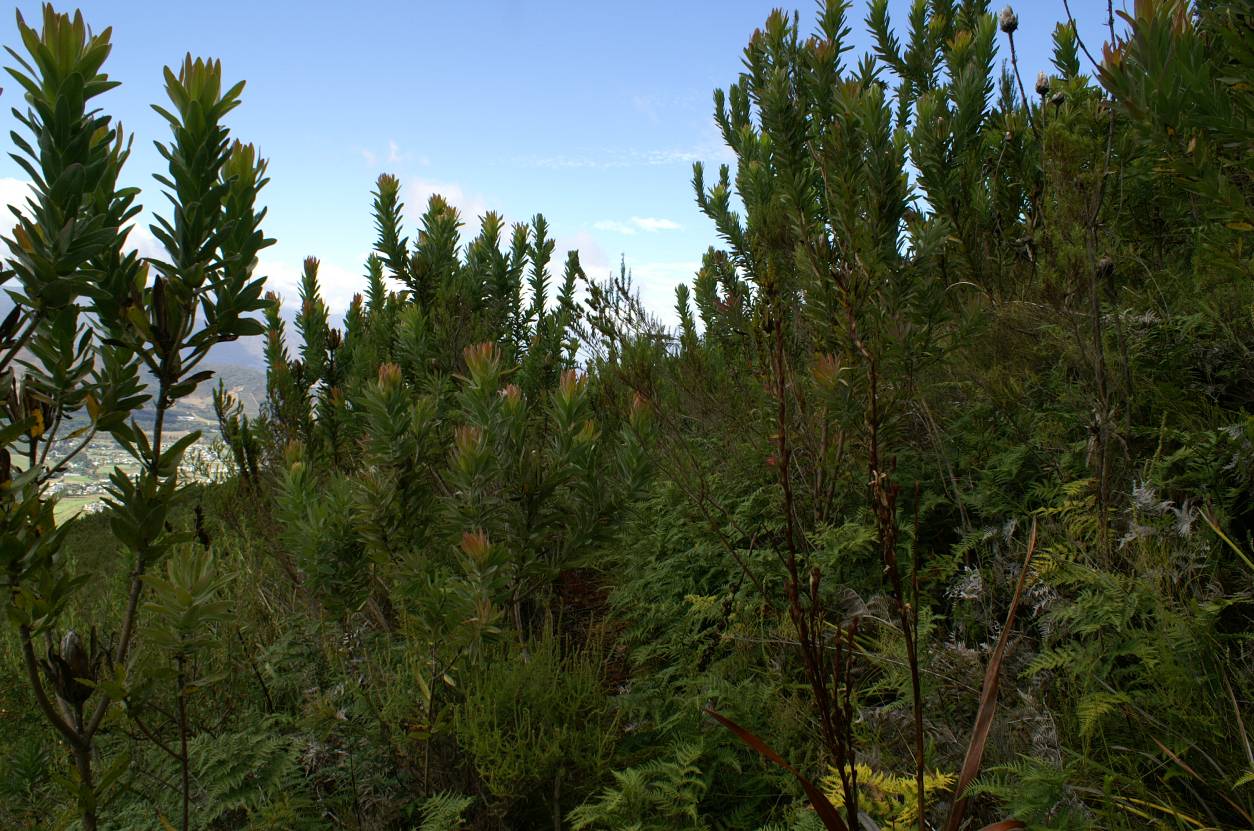
**

**C) Cederberg Wilderness Area: Olifants Sandstone Fynbos**

**
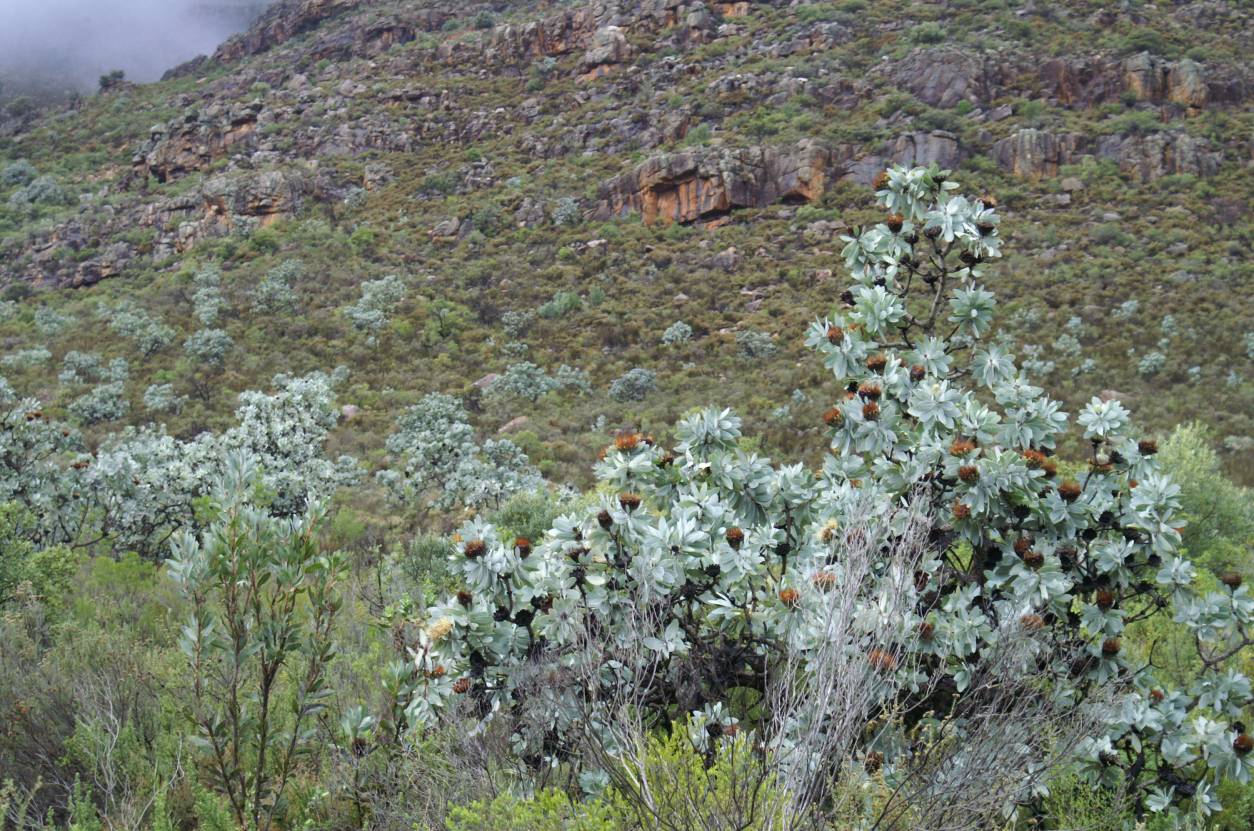
**

**D) Cederberg Wilderness Area: Cederberg Sandstone Fynbos**

**
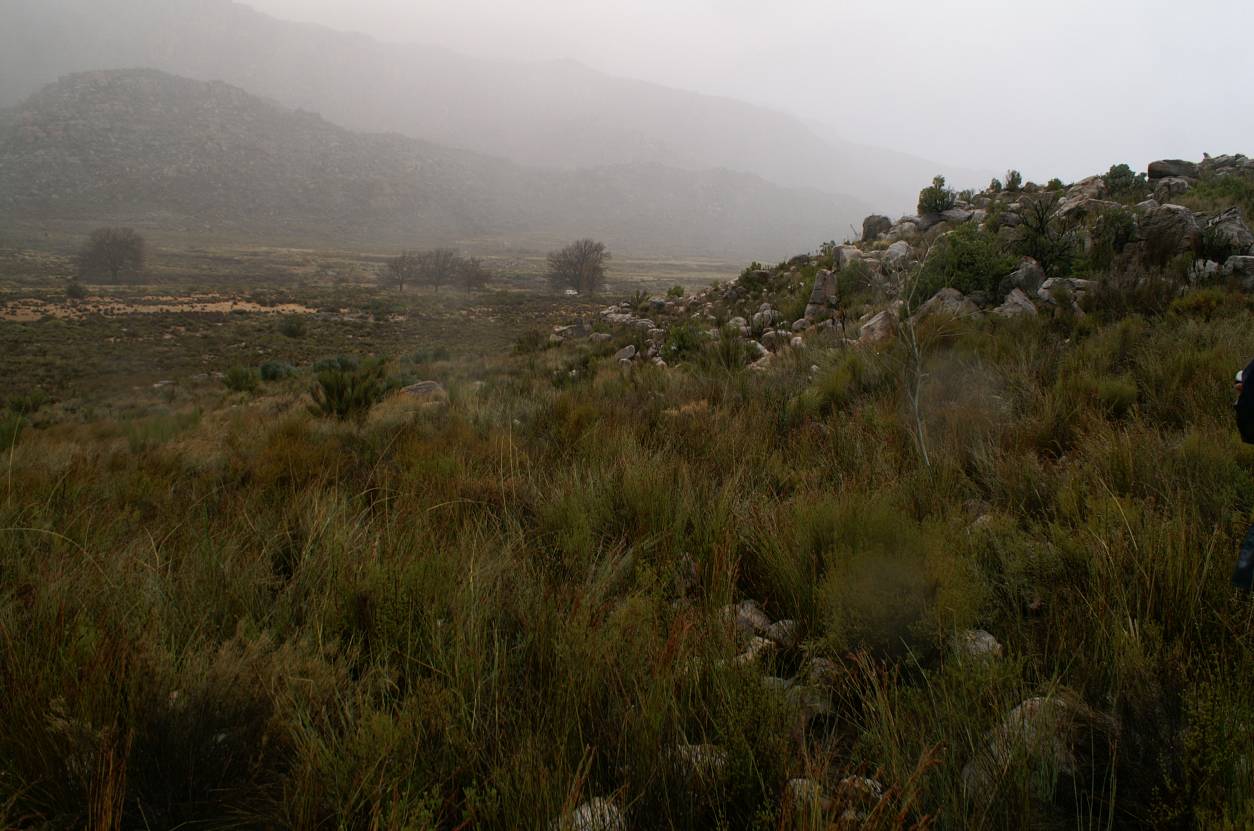
**

**E) Cederberg Wilderness Area: Northern Inland Shale band Vegetation**

**
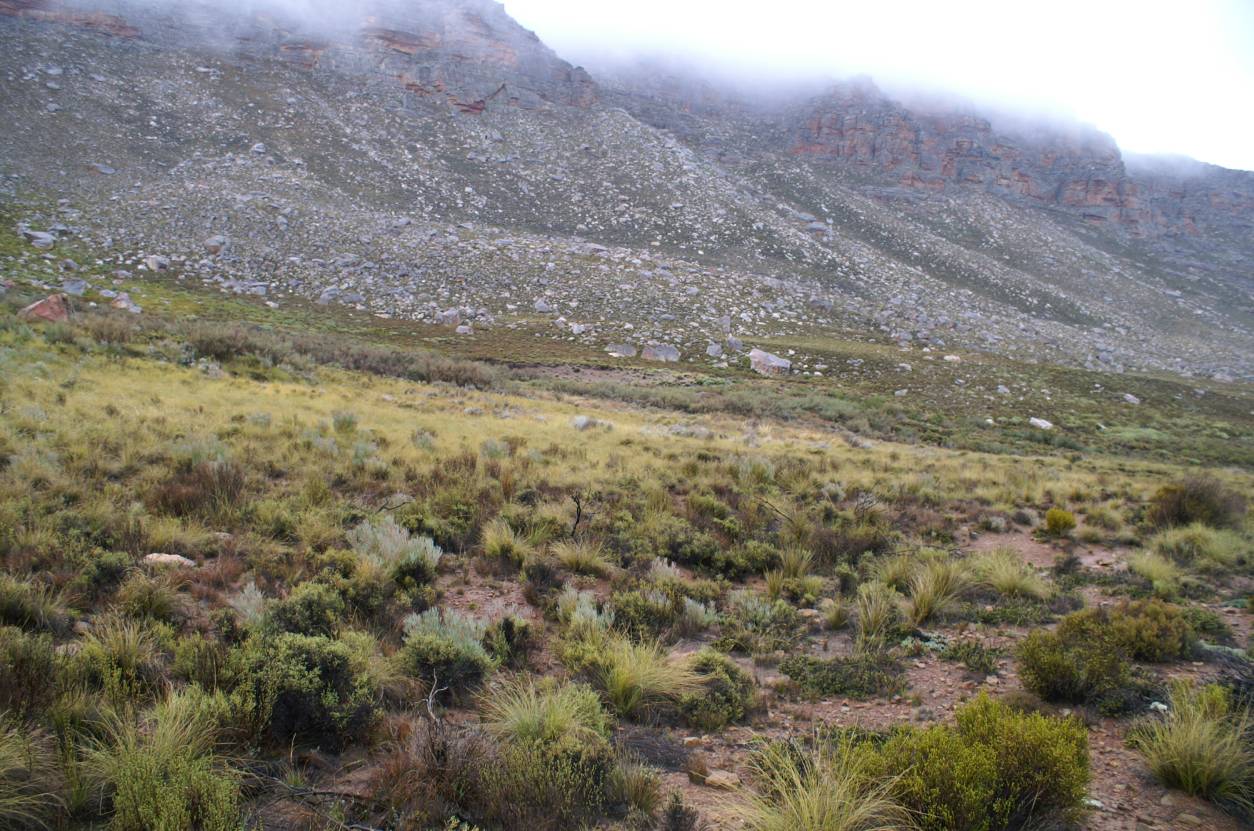
**

**F) Cederberg Wilderness Area: Western Altimontane Sandstone Fynbos**

**
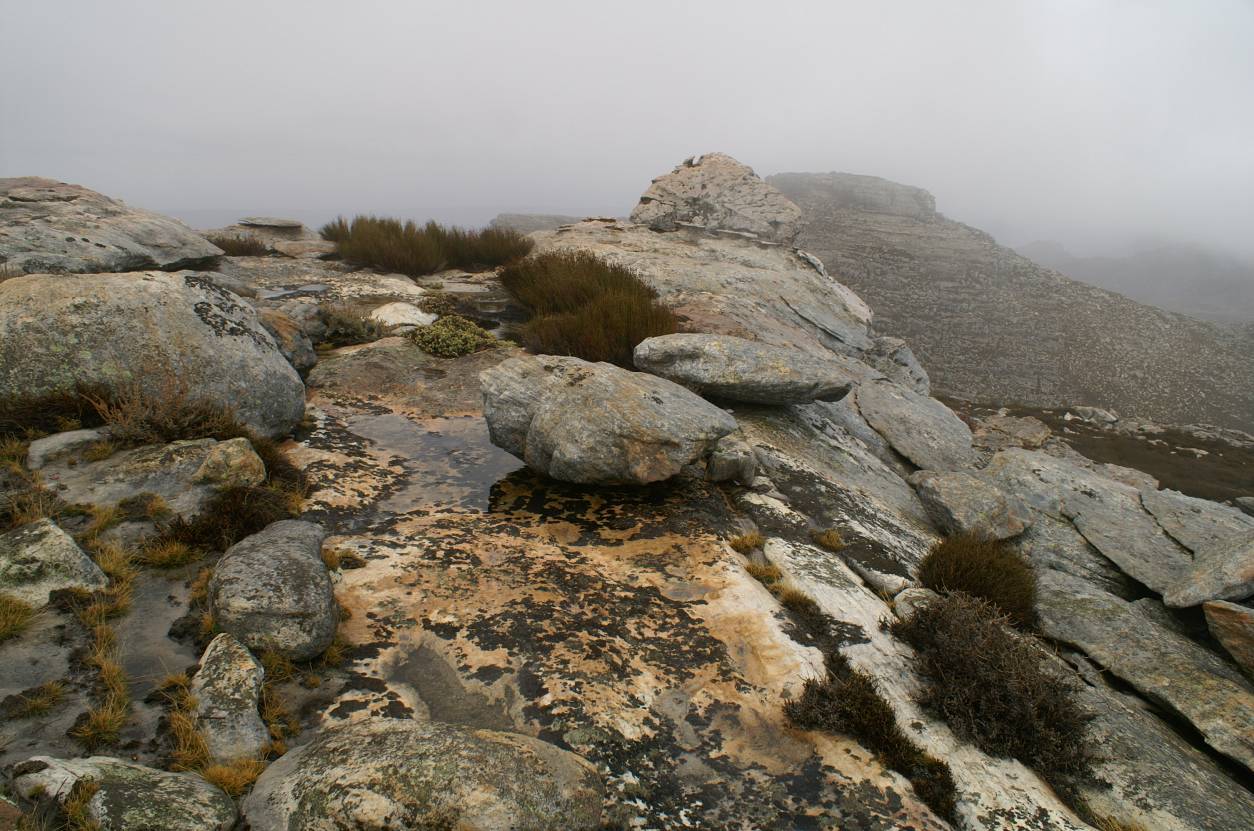
**

**G) Cape Town: Swartland Shale Renosterveld**

**
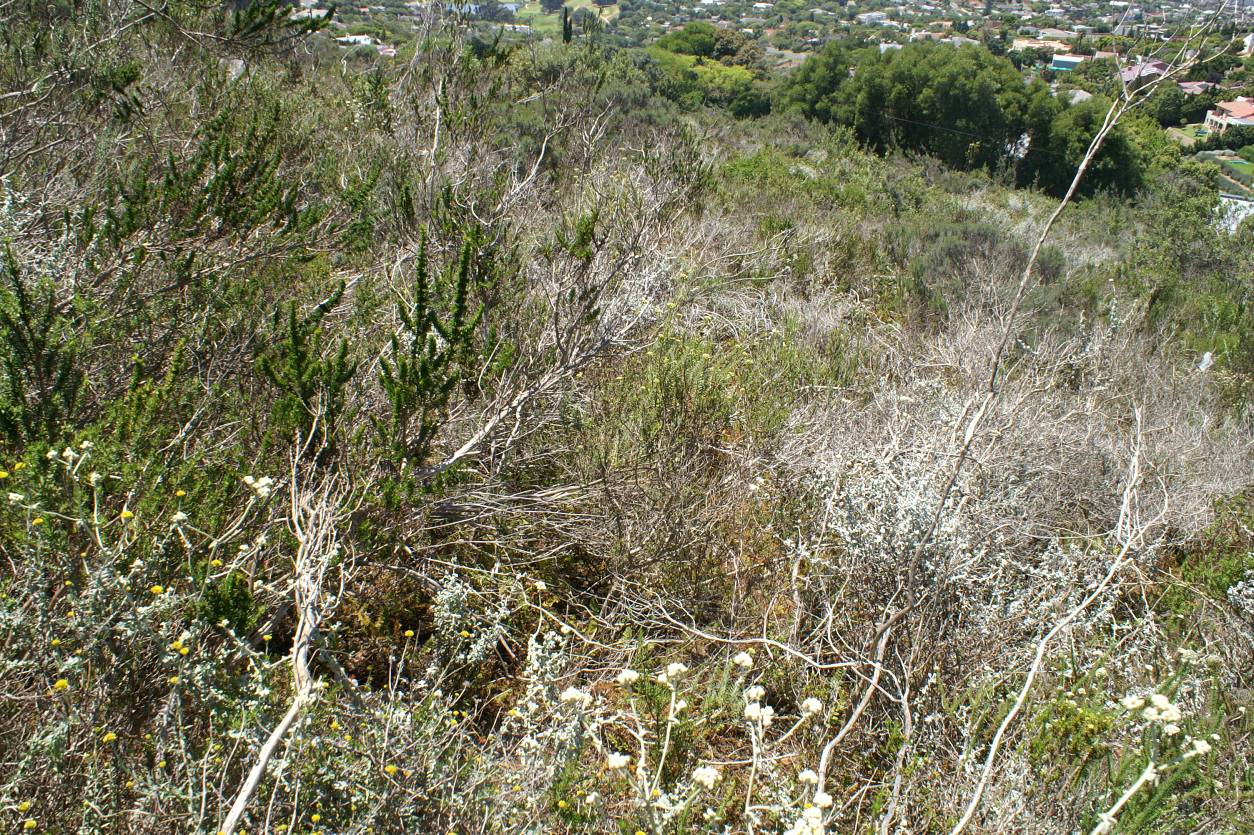
**

**H) Riversdale: Eastern Rûens Shale Renosterveld**

**
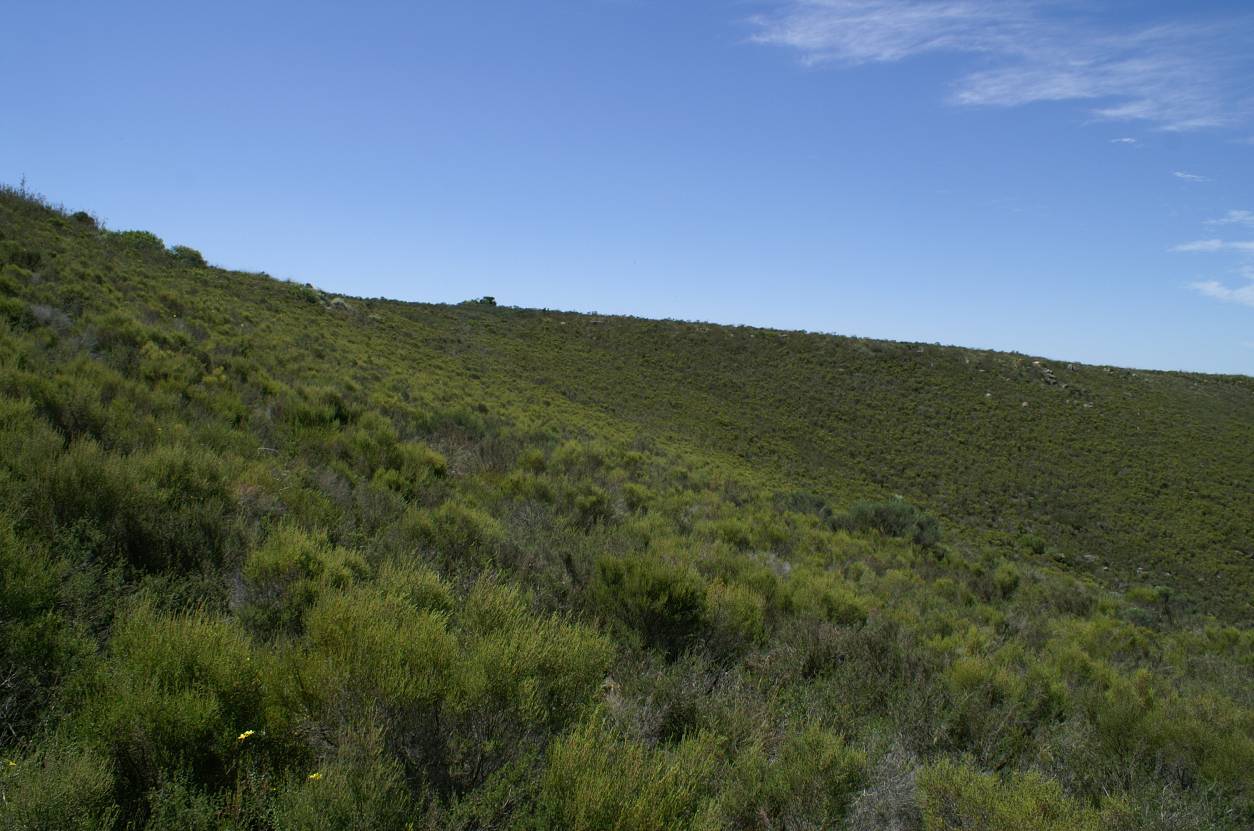
** **
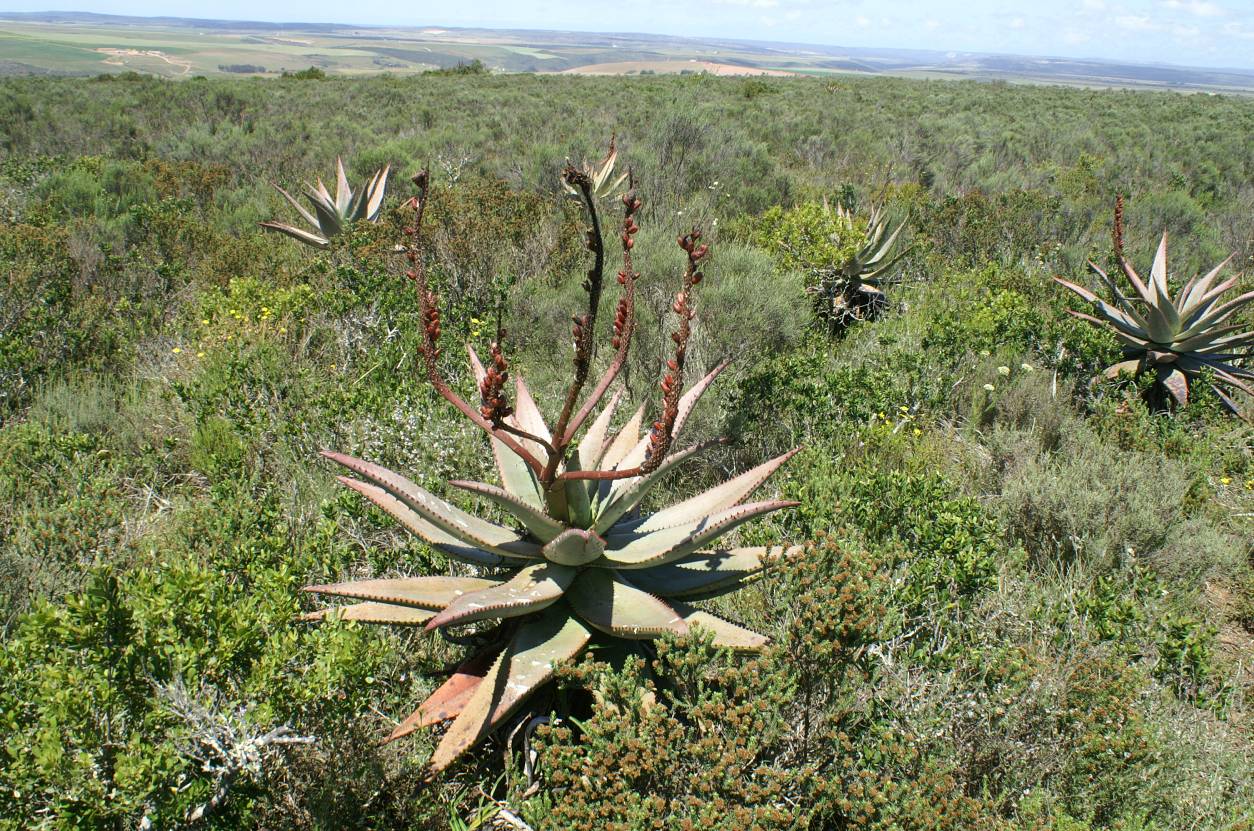
**

**I) Prince Albert: Prince Albert Succulent Karoo**

**
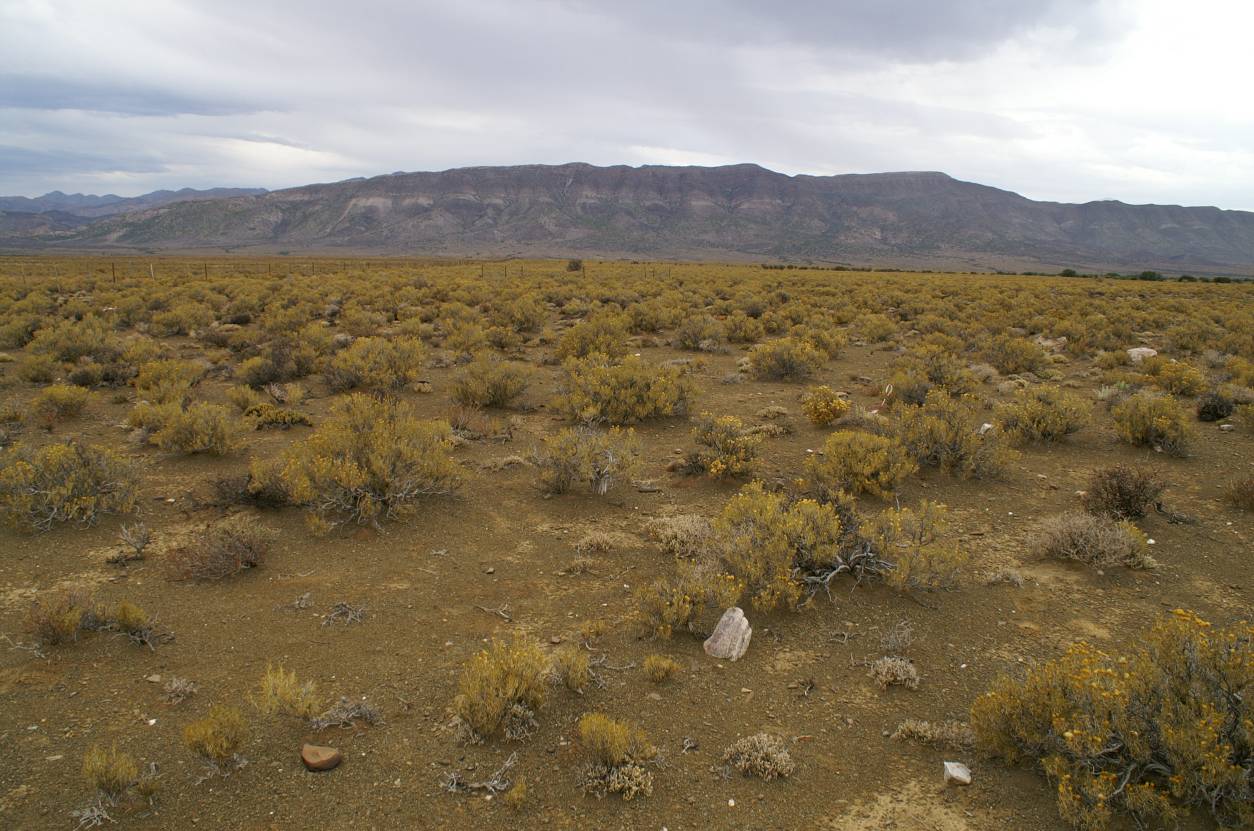
**

**J) Oudtshoorn: Eastern Little Karoo**

**
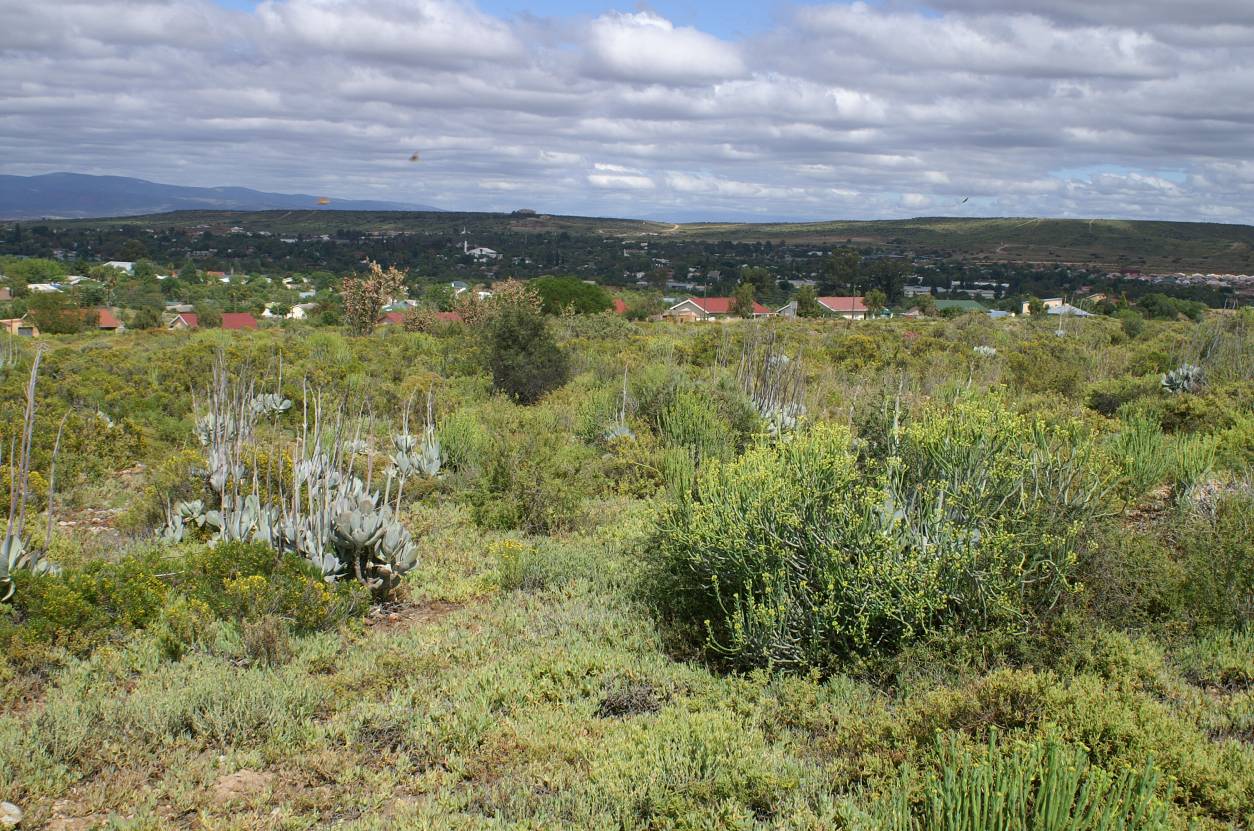
**

**K) Wupperthal: Agter-Sederberg Shrubland**

**
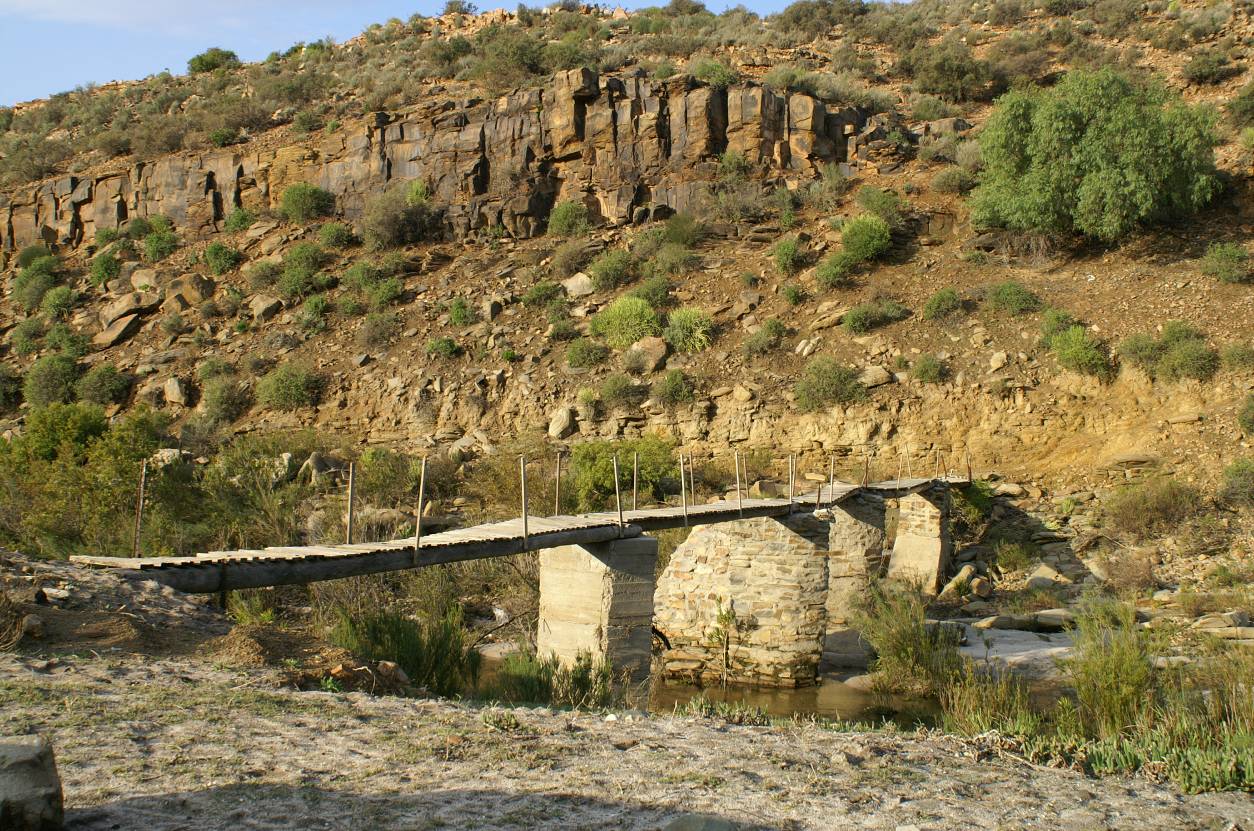
**

**L) Worcester: Robertson Karoo**

**
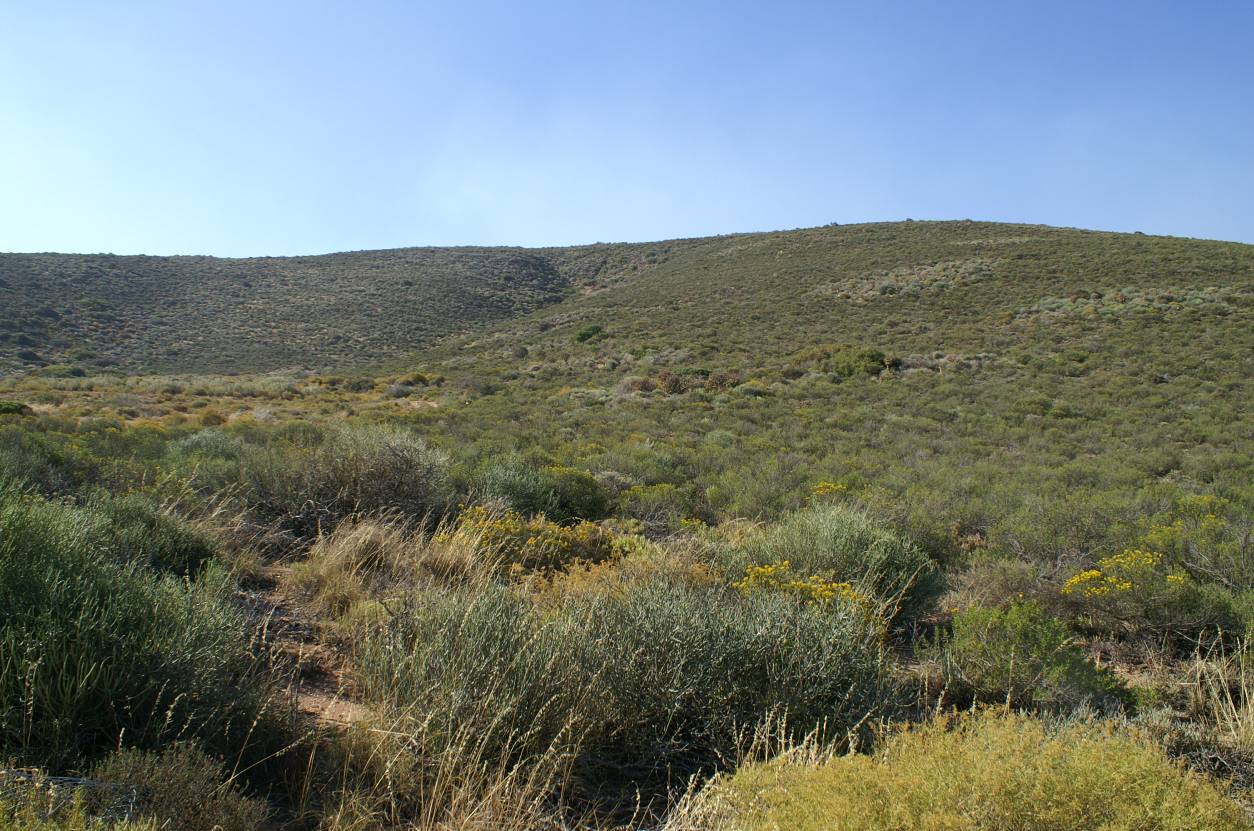

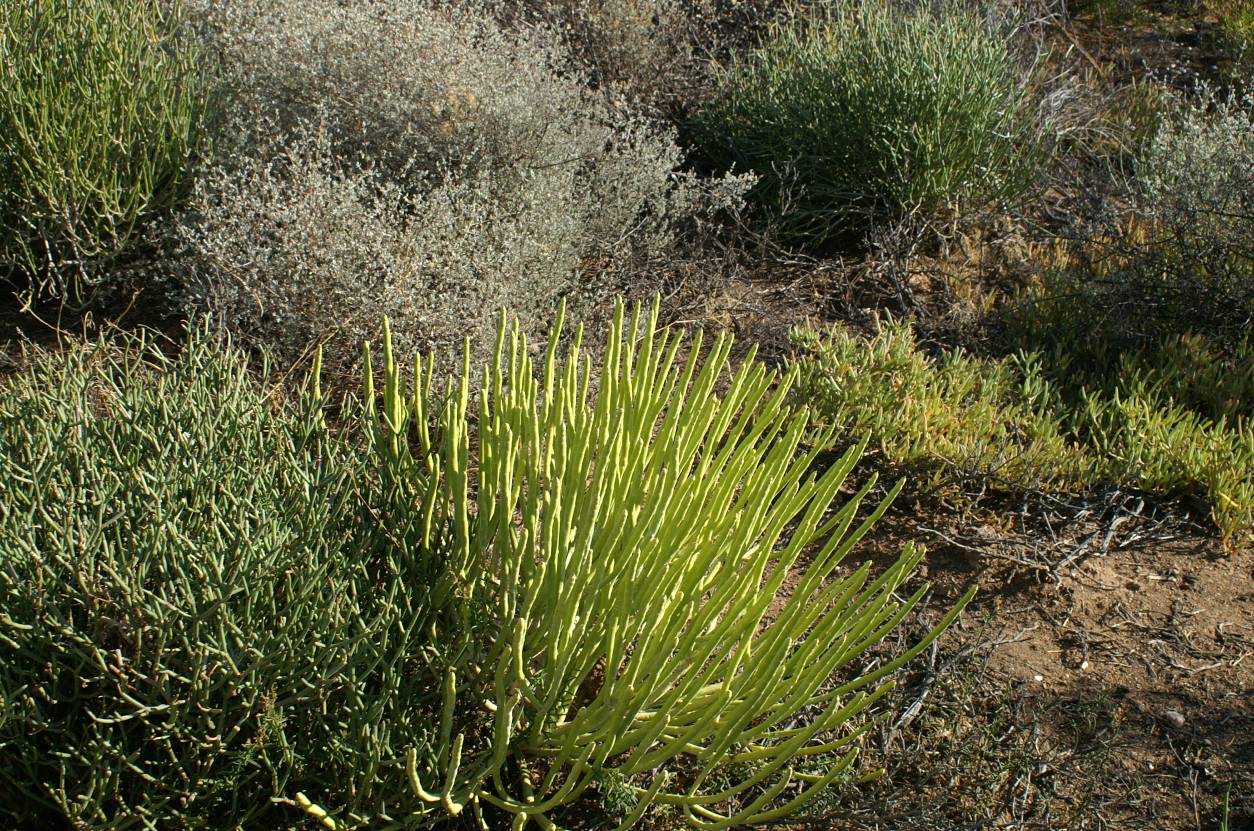
**

**Figure S2. Photos showing examples of vegetation units covered in the study.** Panel numbers match those of the labels in **Figure S1** and **Table S2** in this appendix. Habitat conditions could differ markedly even among sites within the same vegetation unit. Vegetation units are named according to [9].

**Table S2. Information on study sites shown in Figure S1.** Biomes are: FB Fynbos Biome (fynbos, renosterveld and strandveld), NKB Nama-Karoo biome (sites sampled in same way but included in global dataset for sites outside the FB and SKB), and SKB Succulent Karoo Biome. Superscript letters correspond to panel numbers in **Figure S2**.

| **Site** | **Nearest town** | **Latitude** | **Longitude** | **Elevation** | **Biome** |
| --- | --- | --- | --- | --- | --- |
| Bontebok National Park | Swellendam | -34.078417 | 20.467111 | 73 | FB |
| Cape Peninsula, Table Mountain National Park | Cape Town | -34.260111 | 18.393361 | 64 | FB |
| Coast near Steenboksfonteein ^A^ | Lamberts Bay | -32.178033 | 18.314300 | 5 | FB |
| Driehoek ^D^ | Wupperthal | -32.424083 | 19.166167 | 922 | FB |
| Farm Aan Het Berg | Leipoldsville | -32.276633 | 18.529983 | 256 | FB |
| Farm Kapklip | Touwsrivier | -33.298306 | 19.958472 | 874 | FB |
| Farm Sawadee | Clanwilliam | -32.341967 | 18.991517 | 379 | FB |
| Hawequas Scout Farm | Wellington | -33.674056 | 19.058361 | 401 | FB |
| Helderberg Nature Reserve ^B^ | Somerset West | -34.040556 | 18.877889 | 545 | FB |
| Hiking trail between Sneeukop Hut and Wupperthal Site 1 | Wupperthal | -32.339000 | 19.181650 | 1365 | FB |
| Hiking trail between Sneeukop Hut and Wupperthal Site 2 | Wupperthal | -32.335667 | 19.193717 | 1158 | FB |
| Hiking trail between Sneeukop Hut and Wupperthal Site 3 | Wupperthal | -32.327283 | 19.201433 | 965 | FB |
| Jeep track between Welbedacht and Sneeukop Hut Site 1 ^E^ | Wupperthal | -32.459683 | 19.240983 | 1133 | FB |
| Jeep track between Welbedacht and Sneeukop Hut Site 2 | Wupperthal | -32.435000 | 19.232817 | 1337 | FB |
| Jeep track between Welbedacht and Sneeukop Hut Site 3 | Wupperthal | -32.357250 | 19.145883 | 1543 | FB |
| Mont Rochelle Nature Reserve | Franschhoek | -33.903389 | 19.159000 | 755 | FB |
| Moravian Mission land | Genadendal | -34.029972 | 19.558500 | 292 | FB |
| Niewoudt's Pass | Clanwilliam | -32.351117 | 19.006950 | 537 | FB |
| Riviersonderend municipal land | Riviersonderend | -34.137528 | 19.915222 | 208 | FB |
| Rondevlei, Garden Route National Park Site 1 | Sedgefield | -33.993750 | 22.726083 | 18 | FB |
| Rondevlei, Garden Route National Park Site 2 | Sedgefield | -33.989528 | 22.736639 | 26 | FB |
| Sneeukop Hut | Wupperthal | -32.348133 | 19.170217 | 1543 | FB |
| Sneeukop Site 1 | Wupperthal | -32.355167 | 19.148967 | 1687 | FB |
| Sneeukop Site 2 ^F^ | Wupperthal | -32.355083 | 19.161583 | 1926 | FB |
| Sneeukop Site 3 | Wupperthal | -32.354017 | 19.166967 | 1740 | FB |
| Tygerberg Nature Reserve ^G^ | Cape Town | -33.875361 | 18.598750 | 324 | FB |
| Uitkyk Pass ^C^ | Wupperthal | -32.407850 | 19.084650 | 766 | FB |
| Werner Frehse Nature Reserve ^H^ | Riversdale | -34.117250 | 21.248722 | 231 | FB |
| Wolfgat Nature Reserve | Cape Town | -34.070306 | 18.637333 | 12 | FB |
| Karoo National Park Site 1 | Beaufort West | -32.339750 | 22.522056 | 899 | NKB |
| Karoo National Park Site 2 | Beaufort West | -32.329056 | 22.514694 | 991 | NKB |
| Farm Argentina | Prince Albert | -33.165444 | 22.259500 | 745 | SKB |
| Grootkop Nature Reserve ^J^ | Oudshoorn | -33.575694 | 22.216333 | 381 | SKB |
| Hiking trail between Sneeukop Hut and Wupperthal Site 4 ^K^ | Wupperthal | -32.277900 | 19.219350 | 520 | SKB |
| Tierberg Research Station ^I^ | Prince Albert | -33.166278 | 22.268361 | 746 | SKB |
| Department of Agriculture Field Reserve ^L^ | Worcester | -33.621389 | 19.469667 | 278 | SKB |
