# Supplementary material for: The Fynbos and Succulent Karoo Biomes Do Not Have Exceptional Local Ant Richness
Source: PLoS One. 2012 Mar 2;7(3):e31463. doi: 10.1371/journal.pone.0031463 (PMC3292543; doi:10.1371/journal.pone.0031463)
Supplement: Appendix S5 — Semivariograms. (DOC) [file pone.0031463.s006.doc]

**Appendix S5. Semivariograms**

**Supporting Information for:** B. Braschler, S.L. Chown, and K.J. Gaston: The Fynbos and the Succulent Karoo Biomes do not have Exceptional Local Ant Richness

Spatial autocorrelation was not important over the global dataset (**Figure S3**). Considering only our sites in the FB and SK, there was some indication of spatial autocorrelation with sites in the first lag class being more similar to each other in species richness than sites that are more distant from each other (**Figure S4**).

**
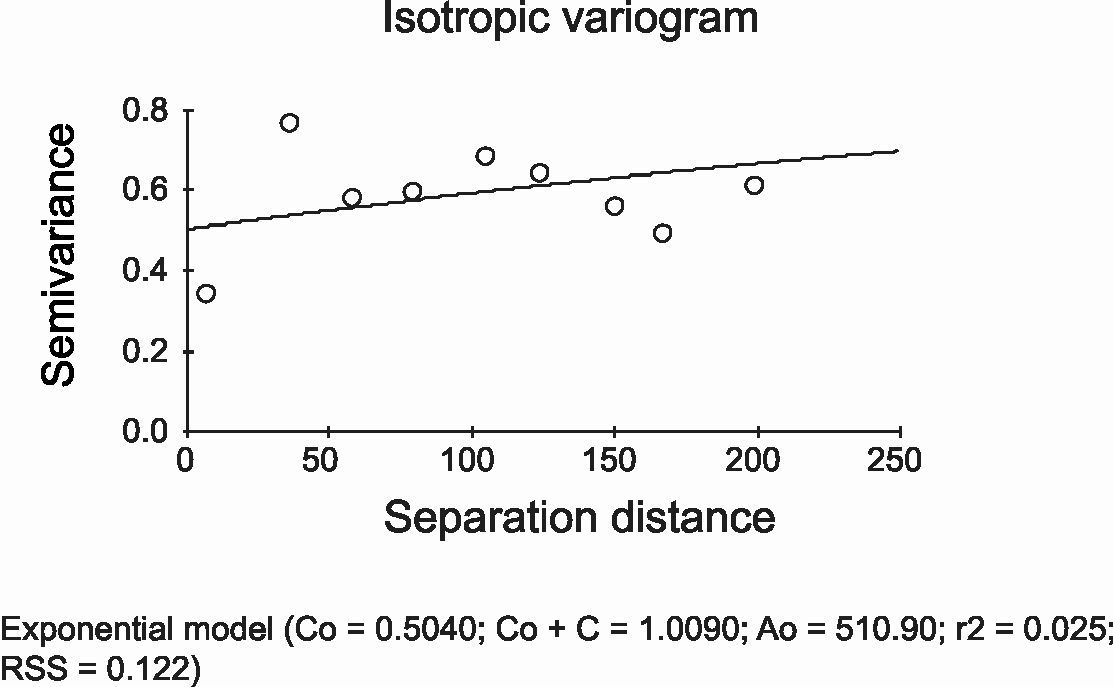
**

**Figure S3. Semivariogram of global dataset on ant species richness as extracted from the literature.**

**Figure S4. Semivariogram of ant species richness in our sites in the FB and SKB.**
