# Supplementary material for: The Fynbos and Succulent Karoo Biomes Do Not Have Exceptional Local Ant Richness
Source: PLoS One. 2012 Mar 2;7(3):e31463. doi: 10.1371/journal.pone.0031463 (PMC3292543; doi:10.1371/journal.pone.0031463)
Supplement: Appendix S6 — Correlation matrices for energy variables and latitude. (DOC) [file pone.0031463.s007.doc]

**Appendix S6. Correlation matrices for energy variables and latitude**

**Supporting Information for:** B. Braschler, S.L. Chown, and K.J. Gaston: The Fynbos and Succulent Karoo Biomes do not have Exceptional Local Ant Richness

Three variables related to energy availability were tested as possible predictors of ant richness both on the regional (FB and SKB) and global scale (database derived from literature excluding our FB and SKB sites). Productive energy was measured as NDVI. Mean annual precipitation represented rainfall which also should influence productive energy. Temperature regulates how well the ants can use the available energy. In addition the effects of latitude were tested as latitude can be expected to be correlated with energy availability. Indeed as shown in **Tables S3** and **S4**, latitude was highly correlated with NDVI, precipitation, and temperature. Among the three energy variables there was a high correlation between mean annual precipitation and NDVI. As the effect of precipitation on ant richness can be expected to be mostly indirect via plant productivity we decided to omit precipitation from our models and focus on NDVI and temperature as, even though they too are correlated, they do represent two independent mechanisms.

**Table S3. Correlations between Latitude and Mean Annual Precipitation, NDVI, and Mean Annual temperature for sites in the FB and SKB. n = 34**

|  |  | **Latitude** | **NDVI** | **MAP** |
| --- | --- | --- | --- | --- |
| **Latitude** | r | 1.00 |  |  |
|  | t |  |  |  |
|  | P |  |  |  |
| **NDVI** | r | -0.71 | 1.00 |  |
|  | t | -5.72 |  |  |
|  | P | <0.0001 |  |  |
| **MAP** | r | -0.59 | 0.72 | 1.00 |
|  | t | -4.13 | 5.90 |  |
|  | P | 0.0002 | <0.0001 |  |
| **Temperature** | r | 0.14 | -0.41 | -0.40 |
|  | t | 0.80 | -2.55 | -2.44 |
|  | P | 0.4289 | 0.0158 | 0.0206 |

**Table S4. Correlations between Latitude and Mean Annual Precipitation, NDVI, and Mean Annual temperature for sites globally excluding the FB and SKB. n = 331**

|  |  | **Latitude** | **NDVI** | **MAP** |
| --- | --- | --- | --- | --- |
| **Latitude** | r | 1.00 |  |  |
|  | t |  |  |  |
|  | P |  |  |  |
| **NDVI** | r | -0.14 |  |  |
|  | t | -2.61 |  |  |
|  | P | -0.0096 |  |  |
| **MAP** | r | -0.27 | 0.75 |  |
|  | t | -5.08 | 20.39 |  |
|  | P | <0.0001 | <0.0001 |  |
| **Temperature** | r | -0.49 | -0.55 | -0.20 |
|  | t | -10.22 | -11.85 | -3.76 |
|  | P | <0.0001 | <0.0001 | <0.0002 |
