# Supplementary material for: The Fynbos and Succulent Karoo Biomes Do Not Have Exceptional Local Ant Richness
Source: PLoS One. 2012 Mar 2;7(3):e31463. doi: 10.1371/journal.pone.0031463 (PMC3292543; doi:10.1371/journal.pone.0031463)
Supplement: Appendix S7 — Regional ant species richness. (DOC) [file pone.0031463.s008.doc]

**Appendix S7. Regional ant species richness**

**Supporting Information for:** B. Braschler, S.L. Chown, and K.J. Gaston: The Fynbos and Succulent Karoo Biomes do not have Exceptional Local Ant Richness

**Table S5. Table giving examples of regional ant species richness outside the Fynbos and Succulent Karoo biomes.** Examples include another South African biome, Mediterranean type habitats and arid habitats, and tropical forests. When area is not given in the literature it is estimated from site maps where available by laying a rectangle or triangle around the sites. Not all information was available for all studies.

| **Location** | **Area** | **Habitat types** | **Number of sites (number of individuals)** | **Collection method** | **Species richness** | **Reference** |
| --- | --- | --- | --- | --- | --- | --- |
| Kruger National Park, South Africa | ~4400 km^2^ | Savanna | 9 (54736) | Pitfall traps | 160 | Parr 2003 |
| Western Australia |  | Open forest (Mediterranean habitat type) | 10 |  | 73 | Majer & Greenslade 1988 |
| Israel |  | Various (transect from Mediterranean coast to the Jordan valley) | 14 |  | 49 | Ofer *et al.* 1978 as seen in Mayer & Greenslade 1988 |
| Israel | ~14500 km^2^ | Sand dunes | 8 | Pitfall traps, direct observations | 18 | Segev 2010 |
| Réserve Spéciale d’Anjanaharibe-Sud and Western Masoala Peninsula, Northern Madagascar | ~125 km distance between reserves | Forest | 8 (24586) | Pitfall traps, litter samples, general collecting. | 325 | Fisher 1998 |
| Ghana | ~94000 km^2^ | Moist tropical forest | 34 (43824) | Litter samples | 176^a^ | Belshaw & Bolton 1994 |
| Monts Doudou, Gabon, Congo Basin |  | Lowland rainforest | 4 | Litter samples, pitfall traps, Malaise traps, Yellow pan traps, sweep netting, beating, general collecting | 310^b^ | Fisher 2004 |
| La Belqique, southeast Cameroon | 40 km^2^ | Secondary lowland rainforest | 7 (243105) | Pitfall traps | 145 | Deblauwe & Dekoninck 2007 |
| Mount Kinabalu, Borneo |  | Tropical rainforest | 10 (7622 in litter, unknown in pitfall traps) | Litter samples, pitfall traps | 283^c^ | Brühl *et al*. 1999 |
| La Selva, Costa Rica | ~15 km^2^ | Lowland rainforest |  | Malaise traps, Berlese samples, litter samples, baits, general collecting | 437^b^ | Longino *et al*. 2002 |
| Viçosa, South-East Brazil |  | Forest fragments | 10 | Litter samples | 138 | Soares *et al*. 2001 |

a includes only strictly leaf litter species; transient arboreal and surface foraging species were excluded from study

b includes arboreal species

c combining several projects using a range of methods in Kinabalu National Park Brühl *et al*. (1998) found 524 species (including arboreal species).

**References cited**

Belshaw, R. & Bolton, B. (1994) A survey of leaf litter ant fauna in Ghana, West Africa (Hymenoptera: Formicidae). *Journal of Hymenoptera Research*, **3**, 5-16.

Brühl, C.A., Gunsalam, G. & Linsenmair, K.E. (1998) Stratification of ants (Hymenoptera, Formicidae) in a primary rain forest in Sabah, Borneo. *Journal of Tropical Ecology*, **14**, 285-297.

Brühl, C.A., Mohamed, M. & Linsenmayr, K.E. (1999) Altitudinal distribution of leaf litter ants along a transect in primary forests on Mount Kinabalu, Sabah, Malaysia. *Journal of Tropical Ecology*, **15**, 265-277.

Deblauwe, I. & Dekoninck, W. (2007) Diversity and distribution of ground-dwelling ants in a lowland rainforest in southeast Cameroon. *Insectes Sociaux*, **54**, 334–342.

Fisher, B.L. (1998) Ant diversity patterns along an elevational gradient in the Réserve Spéciale d’Anjanaharibe-Sud and on the Western Masoala Peninsula, Madagascar. *Fieldiana Zoology* (n.s.), 90:39-67.

Fisher, B. L. (2004) Diversity patterns of ants (Hymenoptera: Formicidae) along an elevational gradient on Monts Doudou in Southwestern Gabon. In: *Monts Doudou, Gabon: a Floral and Faunal Inventory with Reference to Elevational Variation* (Fisher B.L., Ed), California Academy of Sciences, Memoirs 28, California Academy of Sciences, San Francisco. Pp 269-286.

Longino, J.T., Coddington, J. & Colwell, R.K. (2002) The ant fauna of a tropical rain forest: estimating species richness three different ways. *Ecology*, **83**, 689-702.

Ofer, J., Shulov, A. & Noy-Meir, I. (1978) Associations of ant species in Israel: a multivariate analysis. *Israel Journal of Zoology*, **27**, 199-208.

Parr, C.L. (2003) Ant assemblages in a Southern African Savanna: Local processes and conservation implications. PhD Thesis, University of Pretoria, South Africa.

Segev, U. (2010) Regional patterns of ant-species richness in an arid region: The importance of climate and biogeography. *Journal of Arid Environments*, **74**, 646–652.

Soares, S.M., Schoereder, J.H. & DeSouza, O. (2001) Processes involved in species saturation of ground-dwelling ant communities (Hymenoptera, Formicidae). *Austral Ecology*, **26**, 187-192.
